# WI12*_Rhg1_* interacts with DELLAs and mediates soybean cyst nematode resistance through hormone pathways

**DOI:** 10.1111/pbi.13709

**Published:** 2021-10-05

**Authors:** Jia Dong, Matthew E. Hudson

**Affiliations:** ^1^ Department of Crop Sciences University of Illinois Urbana‐Champaign Urbana IL USA

**Keywords:** WI12*_Rhg1_*, DELLA, SCN resistance, hormones

## Abstract

The soybean cyst nematode (SCN) is one of the most important causes of soybean yield loss. The major source of genetic resistance to SCN is the *Rhg1* repeat, a tandem copy number polymorphism of three genes. The roles of these genes are only partially understood. Moreover, nematode populations virulent on *Rhg1*‐carrying soybeans are becoming more common, increasing the need to understand the most successful genetic resistance mechanism. Here, we show that a *Rhg1*‐locus gene (Glyma.18G02270) encoding a wound‐inducible protein (WI12*_Rhg1_*) is needed for SCN resistance. Furthermore, knockout of *WI12_Rhg1_* reduces the expression of *DELLA18*, and the expression of *WI12_Rhg1_* is itself induced by either JA, SA or GA. The content of the defence hormone SA is significantly lower whilst GA_12_ and GA_53_ are increased in *WI12_Rhg1_* knockout roots compared with unedited hairy roots. We find that WI12*_Rhg1_* directly interacts with DELLA18 (Glyma.18G040000) in yeast and plants and that double knockout of *DELLA18* and its homeolog *DELLA11* (Glyma.11G216500) significantly reduces SCN resistance and alters the root morphology. As DELLA proteins are implicated in hormone signalling, we explored the content of defence hormones (JA and SA) in *DELLA* knockout and unedited roots, finding reduced levels of JA and SA after the knockout of *DELLA*. Additionally, the treatment of *DELLA*‐knockout roots with JA or SA rescues SCN resistance lost by the knockout. Meanwhile, the SCN resistance of unedited roots decreases after the treatment with GA, but increases with JA or SA. Our findings highlight the critical roles of WI12*_Rhg1_* and DELLA proteins in SCN resistance through interconnection with hormone signalling.

## Introduction

Soybean cyst nematode (SCN, *Heterodera glycines*) is the most yield‐damaging plant pathogen in the United States. SCN is directly responsible for the yield losses between $1 and 3 billion annually in the United States, a substantial 3%–5% of the overall US farmgate value in soybean production (Allen *et al*., 2017). These losses have driven the development of several technologies aiming to combat SCN and reduce the associated yield loss. Although nematicides have proven to be effective in controlling the SCN population, they have adverse environmental impacts and affect the growth of nearby plants (Sasser and Uzzell, 1991). Nematode‐protectant seed treatment reduces nematode reproduction on plants (Beeman and Tylka, 2018), but the effects of seed treatments on the biology of SCN and root‐zone of plants are not well documented (Beeman and Tylka, 2018). The main means of control is genetic resistance, and the *Rhg1* locus from the SCN resistant soybean plant introduction (PI) 88788 is the main source of resistance available in commercial varieties to minimize the yield loss to SCN in infested fields (Mitchum, 2016). Although this resistance has successfully controlled SCN for decades, field SCN populations gradually overcoming PI 88788‐type *Rhg1* resistance are a major threat to the soybean industry (Mitchum, 2016). Therefore, it is necessary to obtain a deeper understanding of the molecular mechanisms underlying SCN resistance and develop more effective SCN control strategies.

The SCN lifecycle consists of three major stages classified as egg, juvenile, and adult phases (Niblack, 2005). First, second‐stage juvenile nematodes (J2) hatch from eggs in the soil. Then, motile infective J2 nematodes penetrate the root elongation region using their stylet, a hollow spear‐like mouth organ (Davis *et al*., 2008; Davis and Mitchum, 2005). During migration, nematodes enter the root through the epidermis and cortical cells and induce dissolution of root cell walls and cellular rearrangements forming a feeding site called a syncytium (Davis and Mitchum, 2005; Williamson and Hussey, 1996). The nematodes then obtain nutrients from the host plant through the feeding site and continue to grow.

The establishment and maintenance of nematode feeding sites are strongly influenced by the host plant hormone pathways. In the early stages of infection with the nematode *Heterodera schachtii*, methyl jasmonate (MeJA) plays a pivotal role and seems to act as a negative regulator for female development in *Arabidopsis* (Kammerhofer *et al*., 2015). Whilst exogenous application of MeJA to rice has been shown to increase resistance to nematode infection (Nahar *et al*., 2011), there are fewer studies of the effect of GA on nematode resistance. Interestingly, the gibberellin pathway is induced three days after inoculation with a sedentary root‐knot nematode (RKN) (*Meloidogyne graminicola*) in rice (Kyndt *et al*., 2012), suggesting that GA may maintain nematode feeding sites (Kammerhofer *et al*., 2015; Kyndt *et al*., 2012). Recently, a foliar spray with GA was found to increase nematode infection in rice (Yimer *et al*., 2018). Application of salicylic acid (SA) in *Arabidopsis* wild‐type Columbia inhibits cyst nematode parasitism and increases nematode resistance (Wubben *et al*., 2008).

In addition to the direct impact of hormones on nematode infection, hormone‐regulated genes also participate in nematode resistance. DELLA proteins are negative regulators of the GA signalling pathway, participating in plant immune responses to environmental stresses and promoting plant survival (Achard *et al*., 2006; Gomi and Matsuoka, 2003; Jiang and Fu, 2007). By perceiving bioactive GA, the GA receptor GID1 binds to DELLA protein in the nucleus, leading to polyubiquitination of the DELLA protein by E3 ubiquitin‐ligase SCF^SLY1/GID2^ followed by degradation through the 26S proteasome pathway, resulting in various GA‐dependent responses to pathogens (Achard *et al*., 2006; Hou *et al*., 2013; Jiang and Fu, 2007; Ueguchi‐Tanaka *et al*., 2005; Yoshida *et al*., 2014).

To date, few studies have focused on the roles of DELLA and related pathways in plant nematode resistance. A model of gene regulation predicted that a DELLA‐like protein is induced upon soybean RKN attack and is involved in plant immune and stress responses (Beneventi *et al*., 2013). The DELLA protein also works with the JAZ transcription factor from the JA signalling pathway to orchestrate plant growth and defence (Hou *et al*., 2010, 2013). Without GA, DELLA interacts with JAZ, releasing the JAZ‐bound MYC2, which activates the expression of JA‐responsive genes and initiates the defence mode. In contrast, in the presence of GA, DELLA degradation frees JAZ to bind with MYC2, inactivating MYC2, attenuating the JA defence response and activating growth mode (Hou *et al*., 2010, 2013). Elevated JA levels trigger degradation of JAZ to liberate DELLA, resulting in suppressed GA‐mediated plant growth, indicating that JA responds to stress and inhibits growth by fine‐tuning DELLA protein levels (Yang *et al*., 2012).

Three genes (*WI12*, *amino acid transporter* and α*‐SNAP*) located at the *Rhg1* locus contribute to SCN resistance. The function of one of the three, the α‐SNAP*_Rhg1_*, in SCN resistance has been extensively studied (Bayless *et al*., 2016, 2018; Dong *et al*., 2020; Liu *et al*., 2017) and recent efforts have aimed to demonstrate the importance of the *Rhg1* amino acid transporter to SCN resistance (Guo *et al*., 2019). Comparatively, little progress has been made towards understanding the extent to which the WI12*_Rhg1_* protein is involved in SCN resistance, and what its mechanism of action might be.

In this study, we investigate WI12*_Rhg1_* function in SCN resistance. To do so, we first knocked out this protein through CRISPR‐Cas9 genome editing and observed a reduction in SCN resistance in edited roots. To further investigate the molecular function of WI12*_Rhg1_*, a yeast two‐hybrid assay was employed to identify its binding partners. Among several putative binding partners identified, the DELLA18 protein was selected for further study due to its location within a reliable SCN quantitative trait locus (QTL) on the same chromosome as *Rhg1*. We confirmed the interaction between DELLA18 and WI12*_Rhg1_ in planta* using the FRET and BiFC methods and showed that DELLA18 and its homeolog DELLA11 contribute to SCN resistance through CRISPR‐Cas9 genome editing and SCN demographics assays. Furthermore, our study shows that the content of defence hormones JA and SA are significantly lowered in DELLA knockout roots compared to unedited control roots. Although it is known that DELLA acts in GA signalling and is destabilized by GA (Achard *et al*., 2006; Hou *et al*., 2013; Jiang and Fu, 2007; Ueguchi‐Tanaka *et al*., 2005; Yoshida *et al*., 2014), no link has yet been established between GA treatments and SCN resistance. To determine whether GA affects SCN resistance, we treated roots with GA_3_, characterized root architecture, and quantified the development of nematodes in the root, revealing that GA reduces SCN resistance. The GA‐mediated pathway has broad interactions with other hormones such as the defence hormones JA and SA (Fu and Harberd, 2003; Jiang and Fu, 2007; Yimer *et al*., 2018). Strikingly, we found that the treatment with either SA or MeJA recovers the lost SCN resistance of DELLA‐edited roots. Moreover, the application of MeJA onto DELLA‐edited roots inhibits root growth, implying that DELLA acts to modulate SCN resistance and root growth via hormone pathways. To investigate whether WI12*_Rhg1_* responds or contributes to disruptions of DELLA and hormone pathways, we measured the transcripts of *DELLA* and the DELLA‐induced gene *GA20ox1* (Glyma.09G149200) (Cheng *et al*., 2019; Li *et al*., 2018; Taniguchi *et al*., 2018) in WI12*_Rhg1_* knockout roots, finding reduced expression of *GA20ox1* after knockout of either DELLA or WI12*_Rhg1_*. WI12*_Rhg1_* knockout led to decreased SA and increased GA precursor levels in the roots, whilst treatment with JA, SA or GA led to increased expression of WI12*_Rhg1_*. The above results imply that WI12*_Rhg1_* is both responding and contributing to SCN resistance and hormone signalling pathways.

## Results

### The WI12*_Rhg1_* protein contributes to SCN resistance

**Figure 1:**
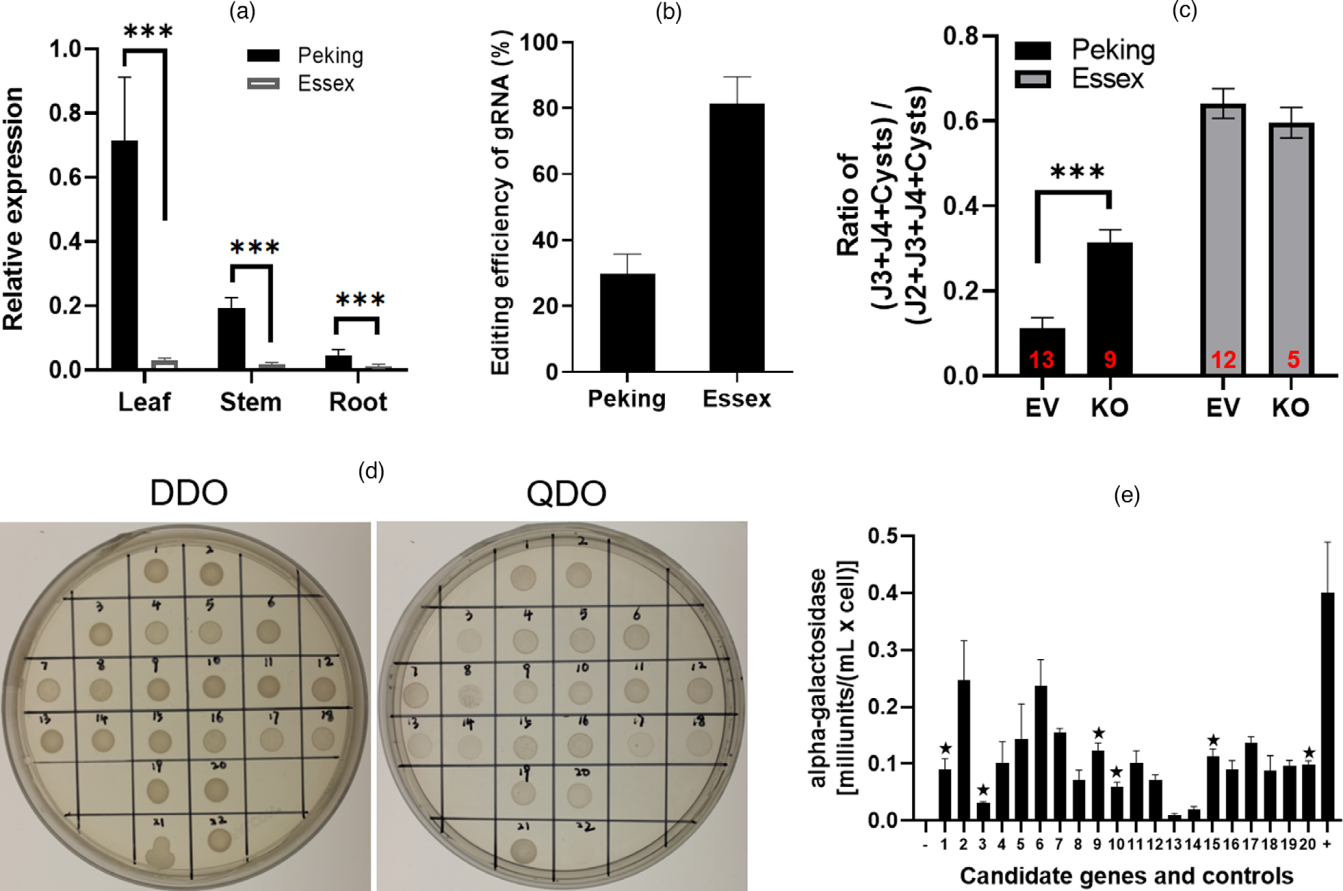
The wound‐inducible (WI12*_Rhg1_*) protein contributes to SCN resistance and binds to multiple protein interactors. (a) RT‐qPCR analysis showing *WI12_Rhg1_* gene expression relative to control at three true‐leaf expanded stages across three tissues (leaf, stem and root) and two soybean varieties (Peking and Essex). Data are means ± standard error (SE) (*n* = 5). (b) gRNA editing efficiency in Peking and Essex hairy roots after CRISPR editing, calculated using ICE analysis. (c) Nematode demographics assay in control (EV) and WI12*_Rhg1_*‐edited (KO) hairy roots in Peking and Essex. Data are means ± SE. The total number of independent edited transgenic hairy roots is displayed at the bottom of each bar. Significance levels are indicated by asterisks: ***P < 0.001; **P < 0.01; *P < 0.05. KO: WI12*_Rhg1_* knockout, EV: empty vector (p201G Cas9 vector without any gRNAs). (d) Yeast two‐hybrid strains plated on plasmid‐selective (DDO) and protein–protein interaction‐selective (QDO) plates. Strains #1–20 hold plasmids encoding candidates for interaction with WI12*_Rhg1_* as described in Table 1, strain #21 is positive control (AtPhyB with AtPIF3) (Ni *et al*., 1999), and strain #22 is negative control (pGADT7 with PGBKT7). DDO: SD (synthetic‐defined)‐Leucine‐Tryptophan; QDO: SD‐Leucine‐Tryptophan‐Histidine‐Adenine. (e) An α‐galactosidase assay is used to quantify the interactions between WI12*_Rhg1_* and candidate proteins. A star above the error bar signifies that the candidate gene is within an SCN QTL, as detailed in Table 2. The *X*‐axis displays negative (−, pGADT7 with PGBKT7) and positive (+, AtPhyB with AtPIF3) controls along with 20 unique candidate genes, Strains #1‐20 as described in detail in Table 1. Data are means ± SE (*n* = 4).

The two wound‐inducible genes *WUN1* and *WI12*, characterized in potato and the halophyte ice plant, respectively, both show tissue‐specific expression (Logemann *et al*., 1988; Yen *et al*., 2001). Therefore, to investigate whether *WI12_Rhg1_* from soybean also exhibits tissue‐specific expression, we performed RT‐qPCR in leaf, stem and root in the SCN‐resistant Peking and SCN‐susceptible Essex varieties (we chose the Peking variety as the proteins interacting with WI12*_Rhg1_* underlie SCN resistance QTL in this variety, as described later in the Result section). Not only did we find that the *WI12_Rhg1_* gene is expressed at significantly higher levels in leaf than in stem and root, but we also found higher expression levels in Peking than in Essex (Figure 1a). To further assess the function of WI12*_Rhg1_* in SCN resistance, we used the CRISPR‐Cas9 system to create deletions in Peking and Essex hairy roots, followed by an SCN demographics assay. SCN susceptibility was significantly increased after knocking out *WI12_Rhg1_* in Peking (Figure 1c). CRISPR‐Cas9 editing efficiency appeared much higher in Essex than in Peking in terms of the percentage of genes carrying a deletion after editing (Figure 1b), likely due to the fact that WI12*_Rhg1_* has three copies in Peking, but only one copy in Essex (Cook *et al*., 2012; Lee *et al*., 2016). Despite the increased editing efficiency, there was no significant effect of deleting the WI12*_Rhg1_* protein in the already susceptible Essex variety (Figure 1b,c). These results demonstrate that the function of WI12*_Rhg1_* is critical to *Rhg1‐*mediated SCN resistance.

### WI12*_Rhg1_* has many potential interactors in yeast

As the WI12*_Rhg1_* protein impacts SCN resistance, we further investigated the molecular function of WI12*_Rhg1_*. A yeast two‐hybrid assay was employed to find potential binding partners of WI12*_Rhg1_*, yielding 92 candidate interactor proteins involved in a diverse range of biological functions (Table S4).

Amongst the 92 candidates, 20 proteins induced rapid growth in the yeast two‐hybrid analysis strains when selected on high‐stringency QDO plates (Table 1), indicating the possibility of a high‐strength interaction with the WI12*_Rhg1_* protein. We next used plate and *α*‐galactosidase assays to characterize and quantify the signal strength of the interactions between these 20 candidate proteins and WI12*_Rhg1_*, finding that more than 2/3 of the candidate proteins exhibited strong interaction with WI12*_Rhg1_* (Figure 1d,e). We further narrowed the number of candidates, to six putative WI12*_Rhg1_*‐binding proteins, by selecting those that are present within the genomic boundaries of the known SCN QTL intervals: These proteins were DELLA18, carbohydrate esterase, ribose‐phosphate pyrophosphokinase, thebaine 6‐O‐demethylase, cysteine protease and Ras‐related protein (Table 2 and Figure 1e). The SCN QTL confidence interval containing the DELLA18 was the smallest amongst the six, and was, thus, more likely to indicate a function of the target gene in SCN resistance than the other five SCN QTLs (Table 2). Additionally, DELLA18 is located on chromosome 18, only 1.6 centimorgans (cM) from the SCN‐resistance locus *Rhg1*. Therefore, we selected DELLA18 for further study as the protein: (1) Has a relatively strong interaction with WI12*_Rhg1_*, (2) is located in the smallest interval amongst the six genes located in SCN QTL, (3) is encoded near the *Rhg1* locus and (4) is a transcription factor involved in the JA and SA pathogen‐defence pathways as well as the pathogen‐related GA pathway.

**Table 1:** Candidate genes selected for further confirmation and quantification

Number	Gene ID	Function of the genes
1	Glyma.18G040000	DELLA
2	Glyma.18G291500	Glucanase‐like protein
3	Glyma.20G129300	Carbohydrate esterase
4	Glyma.08G170100	Elongation factor 2
5	Glyma.03G028800	Methionine aminopeptidase 1
6	Glyma.12G026000	Phosophatehydrolase‐related protein
7	Glyma.04G221300	Superoxide dismutase
8	Glyma.02G264600	COA‐dependent acyltransferase‐related protein
9	Glyma.03G100800	Ribose‐phosphate pyrophosphokinase 5
10	Glyma.08G092800	Thebaine 6‐O‐demethylase
11	Glyma.09G127700	Glucuronosyl transferases
12	Glyma.02G064200	Ribonuclease
13	Glyma.10G242600	3′‐phosphoadenosine 5′‐phosphosulfate synthase
14	Glyma.12G217300	Dehydration‐responsive protein
15	Glyma.15G177800	Cysteine protease
16	Glyma.12G065600	Ras‐related protein Rab‐8A (RAB8A, MEL)
17	Glyma.15G131400	Stress up‐regulated Nod 19 (SURNod19)
18	Glyma.02G262500	Ferritin heavy chain protein
19	Glyma.12G217400	Dehydration responsive protein
20	Glyma.05G115000	Ras‐related protein

**Table 2:** Position of candidate genes relative to SCN QTL

Candidate genes	Description from phytozome	Chromosomal location	SCN QTL on the genome	Linkage group	Relation with SCN QTL
Glyma.18G040000	DELLA	3278869–3281985	SCN‐44‐3 1696616–3474294	G	In QTL (Jiao *et al*., 2015)
Glyma.20G129300	Carbohydrate esterase	37056716–37059915	SCN 34‐2 36575544–40623814	I	In QTL (Winter *et al*., 2007)
Glyma.03G100800	Ribose‐phosphate pyrophosphokinase 5	28980378–28988564	SCN 44‐15 7805399–44176490	N	In QTL (Jiao *et al*., 2015)
Glyma.08G092800	Thebaine 6‐O‐demethylase (redox reaction)	7027499–7030024	SCN 33‐2 3993698–8223512	A2	In QTL (Guo *et al*., 2006)
Glyma.15G177800	Cysteine protease	16892475–16897664	SCN 21‐3 14779070–34757105	E	In QTL (Yue *et al*., 2001)
Glyma.05G115000	Ras‐related protein	30513287–30516952	SCN 18‐1 14845260–34385798	A1	In QTL (Yue *et al*., 2001)

### WI12*_Rhg1_* regulates expression of DELLA and hormone signalling pathways

**Figure 2:**
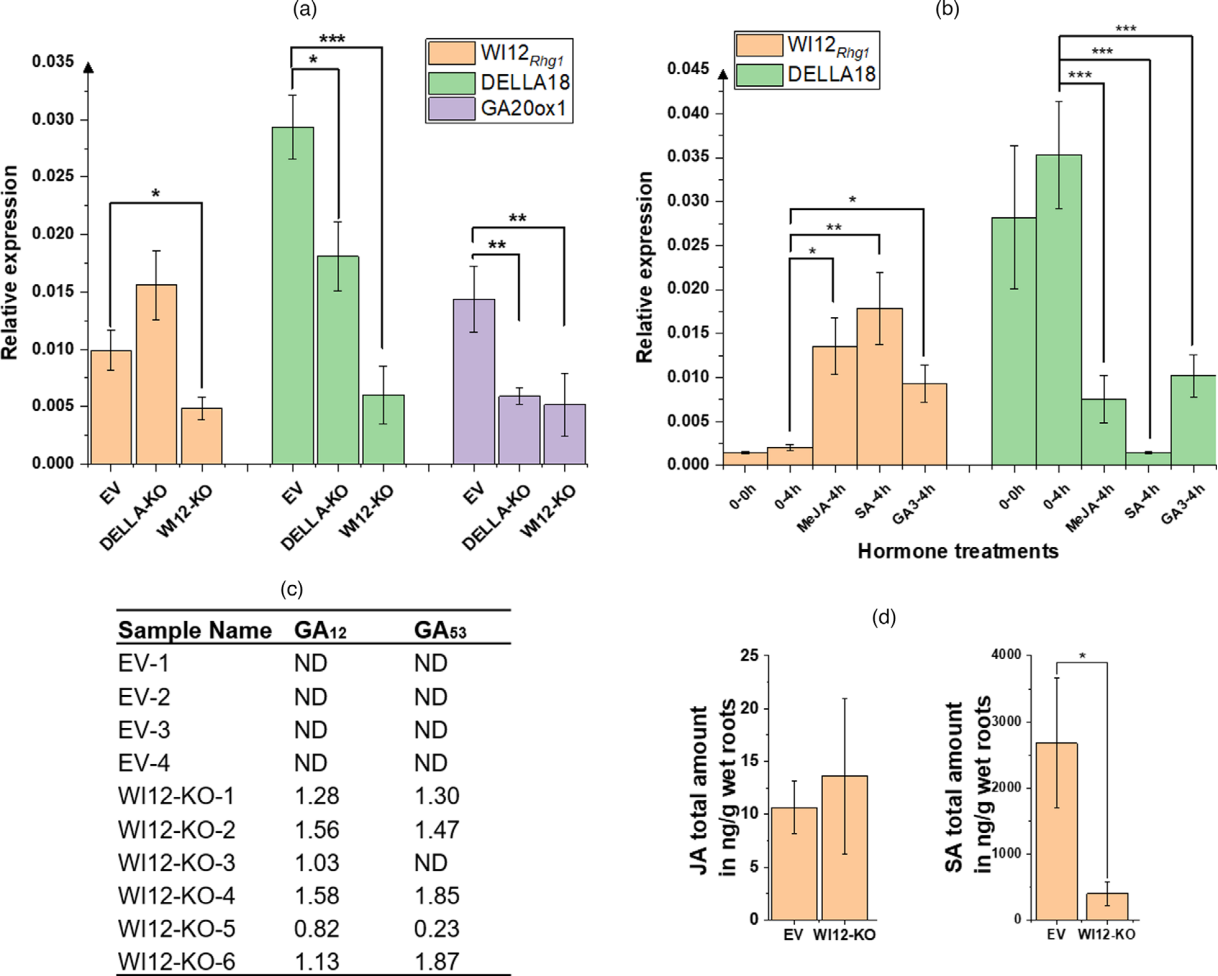
*WI12_Rhg1_* participates in DELLA and hormone pathways. (a) The expression relative to a ubiquitin control gene of *WI12_Rhg1_*, *GA20ox1* and *DELLA18* in Peking unedited hairy roots (EV), DELLA‐knockout (DELLA‐KO) and *WI12* *_Rhg1_*‐knockout roots (WI12‐KO). Data are means ± SE (*n* = 5). (b) The expression relative to control of *WI12_Rhg1_* and *DELLA18* in Peking unedited hairy roots treated with and without GA_3_, MeJA or SA. 0–0h: Zero timepoint controls; 0–4h: Control roots collected after 4 h of water treatment; GA3‐4h: roots collected after 4 h of 10 μm GA_3_ treatment; MeJA‐4h: roots collected after 4 h of 150 μm MeJA treatment; SA‐4h: roots collected after 4 h of 1 mm SA treatment. Data are means ± SE (*n* = 5). Significance levels are indicated by asterisks: ***P < 0.001; **P < 0.01; *P < 0.05. (c)**,** GA_12_ and GA_53_ content in EV and WI12*_Rhg1_*‐edited (WI12‐KO) Peking hairy roots. Data are means ± SE (*n* = 6). (d) JA and SA content in EV and *WI12* *_Rhg1_*‐edited ((WI12‐KO) Peking hairy roots. EV: pCas9 vector without any gRNAs. Data are means ± SE (*n* = 6).

WI12*_Rhg1_* interacts with DELLA, a known regulator of GA and JA hormone signalling (Hou *et al*., 2013; Jiang and Fu, 2007; Yang *et al*., 2012), and as such is likely to regulate the expression of these signalling mechanisms. Therefore, we next aimed to determine whether knockout of WI12*_Rhg1_* or DELLA impacts the expression of each other or the expression of a GA biosynthesis component. *Glyma.09G149200* encodes a predicted GA20ox1, an enzyme catalyzing the final steps of GA synthesis and known to be induced by DELLA (Zentella *et al*., 2007). Knockout of DELLA reduced expression of *Glyma.09G149200* mRNA, which is consistent with prior reports that the expression of *GA20ox* genes is significantly reduced in *Arabidopsis* variants with DELLA mutations (Rieu *et al*., 2008). Expression of *DELLA18* was significantly reduced after knockout of *WI12* *_*Rhg1*_*, indicating that the expression of *DELLA18* is positively regulated by WI12*_Rhg1_*. Knockout of *WI12* *_*Rhg1*_* led to a significant reduction in Glyma.09G149200 expression, showing that WI12*_Rhg1_* impacts the expression of genes regulated by DELLA (Figure 2a).

We next investigated the effects of hormone treatments on the expression of *WI12_Rhg1_* and *DELLA18*. After 4 h of treatment with either GA_3_, SA or MeJA, the expression of *DELLA18* significantly decreased, whereas the expression of *WI12_Rhg1_* significantly increased (Figure 2b). In sum, *WI12_Rhg1_* and *DELLA18* respond to hormone treatments and affect the expression of genes relevant to hormone signalling. We then assayed the content of JA, GA_12_, GA_53_ and SA in WI12*_Rhg1_* knockout roots and unedited roots. In comparison to unedited roots, the content of SA decreased in the WI12*_Rhg1_* knockout roots, but GA_12_ and GA_53_ both increased (Figure 2c,d), indicating that WI12*_Rhg1_* regulates the level of both SA and GA, possibly via DELLA18.

### WI12*_Rhg1_* and DELLA18 proteins interact in yeast and plants

**Figure 3:**
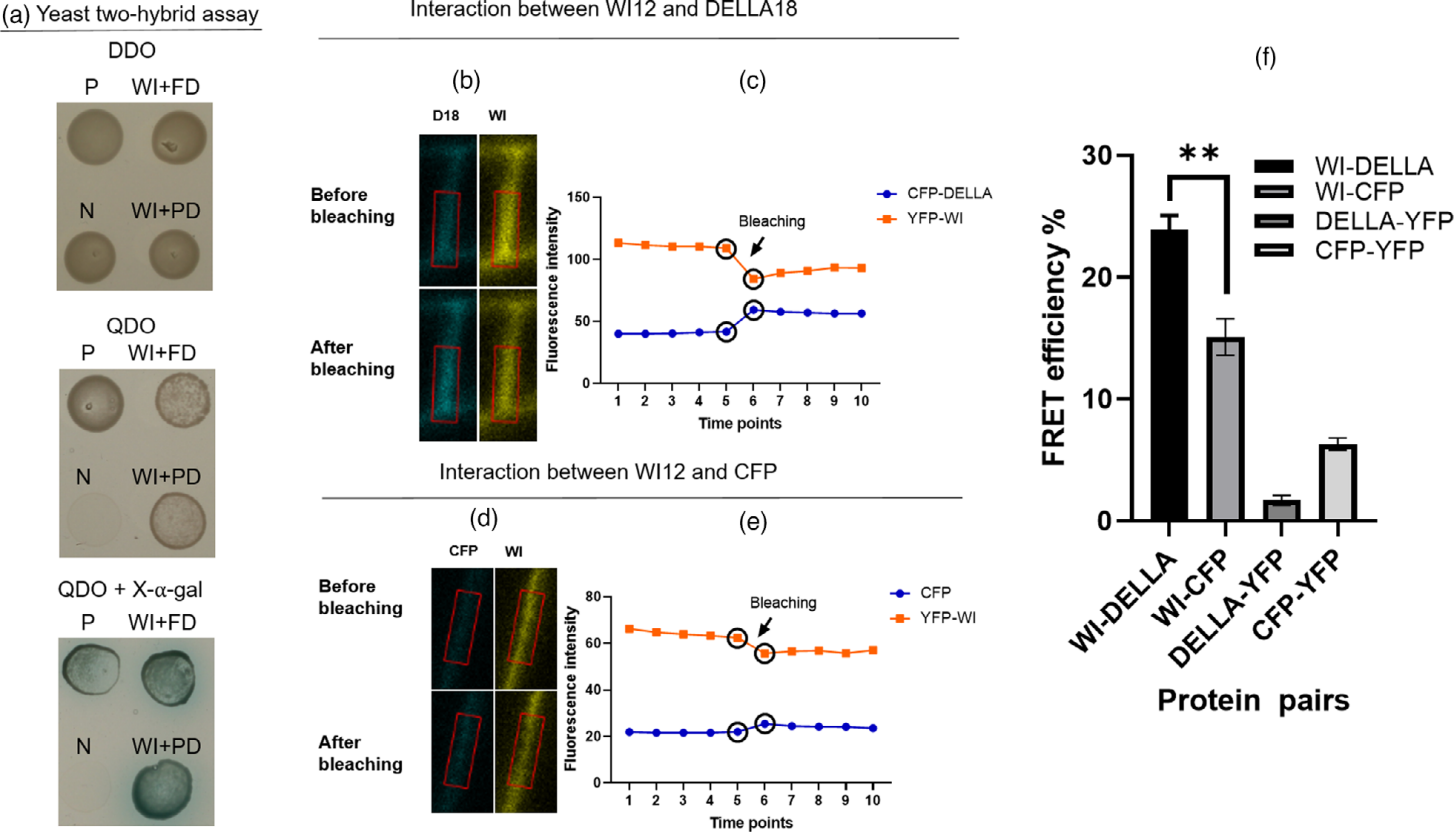
WI12*_Rhg1_* interacts with DELLA18 in yeast and soybean roots. (a) Confirmation of the interaction between WI12*_Rhg1_* protein and DELLA18 in yeast using a yeast two‐hybrid assay. Yeast spot assays were performed on plasmid‐selective DDO (SD‐Leu‐Trp), interaction‐selective QDO (SD‐Leu‐Trp‐His‐Ade), and QDO with X‐α‐galactosidase plates. Each assay was performed using positive control (P: AtPhyB with AtPIF3) and negative control (N: pGADT7 with PGBKT7) strains along with experimental strains WI12*_Rhg1_* with FDELLA18 (WI+FD) and WI12*_Rhg1_* with PDELLA18 (WI+PD). (b–f) Confirmation of WI12 *_Rhg1_*‐DELLA18 interaction in soybean roots using FRET acceptor photobleaching. (b) Fluorescence micrographs show fluorescence brightness changes in CFP (D18: pSM101‐*CFP‐PDELLA18*) and YFP (WI: pSM101*‐YFP‐WI12*) channels directly before and after bleaching, at the fifth and sixth time points, respectively. (c) CFP (pSM101‐*CFP‐PDELLA18*) and YFP (pSM101‐*YFP‐WI12*) fluorescence intensity throughout a photobleaching event occurring between the fifth and sixth time points, as labelled. A decrease in YFP acceptor fluorescence is associated with a significant increase in CFP donor fluorescence, indicating the interaction between PDELLA18 and WI12*_Rhg1_*. (d) Fluorescence micrographs show the fluorescence brightness significantly changes in YFP (WI: pSM101‐*YFP‐WI12*) channel directly before and after bleaching, at the fifth and sixth time points, respectively. (e) CFP (pSM101‐*CFP*) and YFP (pSM101‐*YFP‐WI12*) fluorescence intensity throughout a photobleaching event, occurring between the fifth and sixth time points, as labelled. A decrease in YFP fluorescence is associated with a slight increase in CFP fluorescence. Photobleaching was initiated and ended at the fifth and sixth time points, respectively, as indicated with a circle. Fluorescence intensities were calculated based on the ROI (region of interest) labelled with a red box. (f) FRET efficiency comparison between four protein pairs shows PDELLA18‐WI12*_Rhg1_* interaction. WI‐DELLA: pSM101‐*YFP‐WI12* and pSM101‐*CFP‐PDELLA18*; WI‐CFP: pSM101‐*YFP‐WI12* and pSM101‐*CFP*; DELLA‐YFP: pSM101‐*CFP‐PDELLA18* and pSM101‐*YFP*; CFP‐YFP: pSM101‐*CFP* and pSM101‐*YFP*. Average FRET efficiencies were calculated from three independent experiments. Each experiment consisted of 10 cells.

As our yeast two‐hybrid library screening method isolated only a partial clone of the *DELLA18* cDNA, we next sought to determine whether the full‐length DELLA protein would still interact with WI12*_Rhg1_*. As the Peking‐type *DELLA18* and Fayette‐type *DELLA18* sequences encode distinct proteins (PDELLA18 and FDELLA18), we individually cloned the full‐length cDNA of the *PDELLA18* and *FDELLA18* genes into the prey vector pGADT7‐rec, finding that the full‐length PDELLA18 and FDELLA18 proteins both strongly interact with the WI12*_Rhg1_* protein in the yeast (Figure 3a).

As yeast two‐hybrid assays can identify putative interactions which might not occur in plants, we next employed BiFC and FRET acceptor photobleaching assays to verify the WI12—DELLA18 interaction *in planta*. For the BiFC assay, parent vector NmVen210:*WI12–X*:CVen210 showed a weaker fluorescent signal in both tobacco epidermal and Peking hairy root cells (Figure S6). However, vectors NmVen210:*WI12–PDELLA18:*CVen210 and NmVen210:*WI12–FDELLA18*:CVen210 produced BiFC signals in both tobacco epidermal and hairy root cells (Figure S6), demonstrating that WI12*_Rhg1_* interacts with both PDELLA18 and FDELLA18 in plant cells.

We next constructed vectors fusing *PDELLA18* and *WI12_Rhg1_* to ORFs encoding full‐length fluorescent proteins: *PDELLA18* was fused with *CFP* in plasmid pSM101*‐CFP‐PDELLA18*, whilst the *WI2_Rhg1_* gene was fused with *YFP* in plasmid pSM101*‐YFP‐WI12*. The plasmids were then individually transformed into tobacco leaf or soybean hairy root cells and imaged to confirm fluorescent signals and determine the subcellular localization of these proteins. In comparison with membrane‐ and nuclear‐targeted fluorescent controls 51V (pSM101‐*YFP‐51V*) and 41V (pSM101‐*YFP‐41V*), respectively, we found that the WI12*_Rhg1_*‐YFP protein displays strong fluorescence in the perinuclear region and cell periphery, whilst PDELLA18‐CFP generates fluorescent signals in the nucleus and cell periphery (Figures S7 and S8).

After confirming the presence of fluorescent signals from both the proteins of WI12*_Rhg1_* and PDELLA18 during subcellular localization assays, the pSM101*‐CFP‐PDELLA18* and pSM101*‐YFP‐WI12* plasmids were then used in a FRET acceptor photobleaching assay as a secondary method of verifying protein–protein interactions *in planta*. Soybean hairy root cells harbouring the pSM101*‐CFP‐PDELLA18* and pSM101*‐YFP‐WI12* plasmids provide further evidence for interaction between WI12_Rhg1_ and PDELLA18 by showing an increase in CFP fluorescence intensity after photobleaching the acceptor YFP (Figure 3b,c). In contrast, control images did not show a substantial increase in CFP fluorescence after photobleaching of acceptors (Figure 3d,e, Figure S9). Because the FRET efficiency for the WI12‐DELLA sample is significantly higher than the controls, the WI12*_Rhg1_* protein directly interacts with DELLA18 in Peking root cells (Figure 3f).

### DELLA proteins contribute to SCN resistance and influence the defence hormone responses

Proteins from the DELLA family are known to play a vital role in plant growth and defence in *Arabidopsis* and rice (Hou *et al*., 2010; Yang *et al*., 2012), but their effect on SCN resistance and soybean root growth has not been documented.

**Figure 4:**
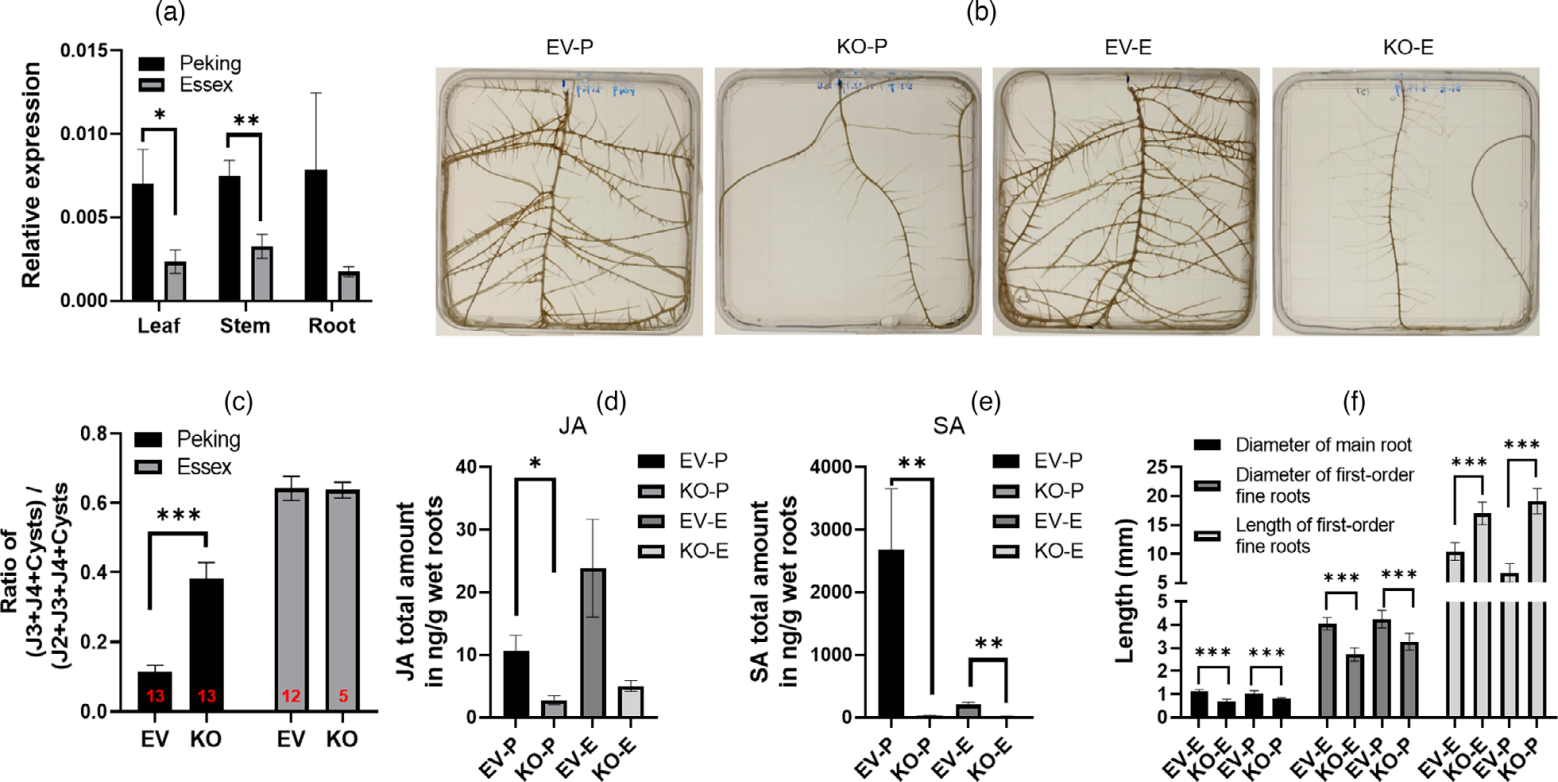
DELLA affects hormone content and root architecture, and is critical for resistance to nematode infection. (a) Investigation of *DELLA* expression relative to a control gene across various soybean tissues (leaf, stem and root) and varieties (Peking and Essex) using RT‐qPCR. Data are means ± SE (*n* = 5). (b) Hairy roots grown on ICM agar plates. EV‐P: control (pCas9 vector without any gRNAs) in Peking, KO‐P: DELLA‐knockout in Peking, EV‐E: control (p201G Cas9 vector without any gRNAs) in Essex and KO‐E: DELLA‐knockout in Essex. (c) Nematode demographics assay in control and *DELLA*‐edited Peking and Essex hairy roots. Data are means ± SE. The total number of independent‐edited transgenic hairy roots is displayed in red text at the bottom of each bar. (d) JA content in control and *DELLA*‐edited Peking and Essex hairy roots. Data are means ± SE (*n* = 4). (e) SA content in control and *DELLA*‐edited Peking and Essex hairy roots. Data are means ± SE (*n* = 4). (f) Root measurements in control and *DELLA*‐edited Peking and Essex hairy roots. Data are means ± SE. The total number of independent‐edited transgenic hairy roots is equivalent to the number of nematode demographics assays displayed in Figure c. Significance levels are indicated by asterisks: ***P < 0.001; **P < 0.01; *P < 0.05.

As gene‐sequence variation in different soybean varieties has been associated with SCN resistance (Bayless *et al*., 2016), we sequenced the full‐length cDNAs encoding DELLA from Fayette 99, Peking, Essex and W82. Peking possesses two variants compared to the other three genotypes: a nine‐base pair insertion at nucleotides 26–34 adding three additional alanine residues and a SNP at nucleotide 1245 resulting in no amino acid change (Figure S10). Furthermore, *DELLA18* colocalizes with the SCN 8‐1 QTL (Chang *et al*., 1997) and lateral root density QTL 1‐1 (Williams *et al*., 2012), both identified using parent lines Essex and Forrest (Table S3). *DELLA18* is also encoded within the SCN 44‐3 QTL (Jiao *et al*., 2015), identified using parent lines William82 and PI 437655. Additionally, *DELLA18* is only 1.6 cM away from the *Rhg1* locus and its homeolog *DELLA11* localizes in the SCN 39‐2 QTL (Wu *et al*., 2009) (Table S3). This variation in *DELLA* sequences combined with co‐location with QTL makes it more likely that they are involved in SCN resistance, and indicates that they may play a role in differential responses to SCN in different genotypes of soybean. Using RT‐qPCR, we showed that *DELLA18* mRNA was expressed at higher levels relative to the control gene in SCN‐resistant Peking than in the susceptible Essex variety in root, stem and leaf tissues (Figure 4a).

Therefore, we determined that *DELLA18* was a strong candidate for a gene that may mediate genetic variation in SCN resistance, and employed CRISPR‐Cas9 genome editing to observe the effect of *DELLA* knockouts on root growth and the ability to resist SCN infection. As DELLA18 has a high peptide sequence identity (90.6%) with DELLA11 (Figure S1) and genes encoding both proteins localize within SCN QTL (Table S3), we, therefore, designed gRNAs to target both DELLA coding sequences to ensure a complete loss of DELLA18‐related activity (Figure S11). Knocking out both DELLA proteins significantly increased nematode development on Peking roots (Figure 4c) and decreased the content of both JA and SA (Figure 4d,e). However, GA_1,_ GA_3,_ GA_4_ and GA_12_ were undetectable in both control and DELLA‐knockout roots. Moreover, as the content of SA is much higher in unedited Peking than unedited Essex, it may be possible that SA plays an important role in SCN resistance (Figure 4e). Additionally, *DELLA*‐edited Peking and Essex roots display altered root morphology, with thinner main roots and longer and thinner first‐order fine roots compared to the unedited controls (Figure 4b,f). Thus, the DELLA proteins affect root growth, the content of defence hormones and SCN resistance, suggesting that DELLA affects SCN resistance by regulating the content of JA and SA in roots.

### JA and SA rescue SCN resistance lost through *DELLA* deletion

**Figure 5:**
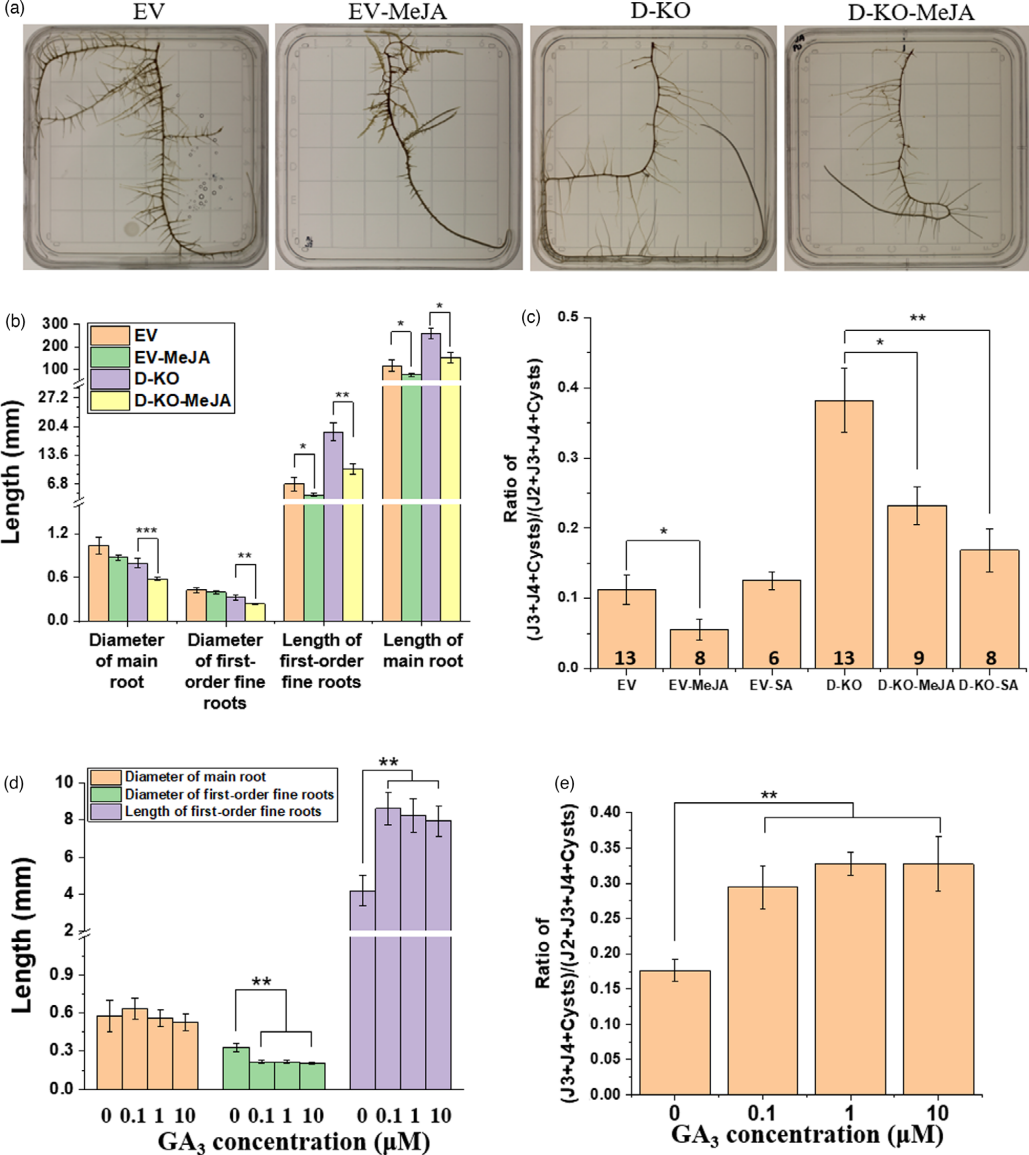
Nematode resistance lost through *DELLA* deletion is partially rescued after treatment with defence hormones, while GA_3_ decreases SCN resistance. (a) Root phenotype images from left to right in order: unedited hairy roots (Peking genotype) with 0 μm MeJA treatment (EV), unedited hairy roots with 150 μm MeJA treatment (EV‐MeJA), *DELLA*‐knockout hairy roots with 0 μm MeJA treatment (D‐KO), *DELLA*‐knockout hairy roots with 150 μm MeJA treatment (D‐KO‐MeJA). (b) MeJA treatments impact root growth. Data are means ± SE. The total number of independent‐edited transgenic hairy roots is equivalent to the number of nematode demographics assays displayed in Figure (c). (c) MeJA or SA treatments impact SCN infection in Peking. Unedited hairy roots with 1 mm SA treatment (EV‐SA); *DELLA*‐knockout hairy roots with 1 mm SA treatment (D‐KO‐SA). Data are means ± SE. The total number of independent‐edited transgenic hairy roots is displayed in the bottom of the bars. (d) Root measurements without (0) and with GA_3_ treatments (0.1, 1, 10 µm). Data are means ± SE (*n* = 6). (e) GA_3_ treatments of unedited hairy roots impact SCN resistance. Data are means ± SE (*n* = 6). Significance levels are indicated by asterisks: ***P < 0.001; **P < 0.01; *P < 0.05.

Previous studies have shown that JA triggers degradation of JAZ1 to liberate DELLA, which inhibits plant growth but promotes a defence response (Hou *et al*., 2010; Yang *et al*., 2012). We found that exogenous application of MeJA onto unedited roots (hairy roots derived from the Peking genotype carrying pCas9 vector without any gRNAs) slightly increased SCN resistance but inhibited root growth. Applying the same treatment to *DELLA*‐edited roots partially rescues the loss of SCN resistance resulting from *DELLA* deletion, but induces growth of smaller roots (Figure 5a,c), which fits well with previously established models of DELLA orchestrating plant growth and defence via the antagonistic JA and GA signalling pathways (Hou *et al*., 2010; de Vleesschauwer *et al*., 2016; Yang *et al*., 2012). Treatment with SA also partially recovers the SCN resistance lost as a result of the knockout of *DELLA* (Figure 5c). The above results indicate that DELLA mediates observed effects on SCN resistance at least partly via JA and SA signalling pathways.

Treatment with GA enhances RKN infection in rice (Hossain *et al*., 2017; Yimer *et al*., 2018). However, it was unknown whether GA affects SCN development in soybean roots. Therefore, we treated unedited roots with three different concentrations of GA_3_. All three GA_3_ treatment concentrations decreased nematode resistance and induced growth of longer and thinner first‐order fine roots, compared to controls not supplied with GA_3_ (Figure 5d,e). However, there was no significant change in root phenotypes or nematode resistance between the three GA_3_ treatment concentrations (Figure 5d,e). Our findings suggest that GA negatively impacts the soybean immune response to SCN infection, but aids in the growth of the first‐order fine roots.

## Discussion

### WI12*_Rhg1_* participates in SCN resistance, DELLA and hormone signaling pathways

The *Rhg1* repeat locus has been extensively studied for its critical role in resistance to SCN infection (Caldwell *et al*., 1960; Cook *et al*., 2012; Cregan *et al*., 1999). Using RNAi gene silencing, all three genes (*WI12*, *α‐SNAP* and amino acid transporter) in the repeated 31.2 kb segment at the *Rhg1* locus were found to contribute to SCN resistance in the Fayette soybean genotype (Cook *et al*., 2012). Subsequently, substantial efforts have aimed to reveal the molecular function of the *α*‐SNAP*_Rhg1_* in SCN resistance (Bayless *et al*., 2016, 2018; Liu *et al*., 2017). In contrast to the *α*‐SNAP*_Rhg1_* gene, molecular mechanisms governing the role of *WI12_Rhg1_* in SCN resistance have yet to be elucidated, and no further demonstration of the role of the *WI12_Rhg1_* gene in SCN resistance has been published. Here, we conclusively demonstrate the involvement of this protein in SCN resistance, and report several findings furthering the understanding of the molecular mechanism of *WI12_Rhg1_* in SCN resistance.

Initially, we found that the *WI12_Rhg1_* mRNA is highly expressed in the SCN resistant Peking variety relative to the SCN susceptible Essex variety. Our results, coupled with prior studies showing that the expression of the wound‐induced cell‐wall protein WI12 is induced by pathogen infection and wound treatment in the ice plant (Yen *et al*., 2001) suggested that WI12*_Rhg1_* may have an important role in SCN infection. Therefore, we deleted the *WI12_Rhg1_* gene in Peking hairy roots and observed an increase in SCN susceptibility in the WI12*_Rhg1_*‐knockout Peking hairy roots (Figure 1c). Moreover, we observed that *WI12_Rhg1_* knockout caused decreased SA levels and increase in levels of the active GA precursors GA_12_ and GA_53_ (Figure 2c,d); other GAs were not present at detectable levels. Following the establishment of the role of WI12*_Rhg1_* in SCN resistance, a yeast two‐hybrid assay was employed to identify a number of potential binding partners of the WI12*_Rhg1_* protein (Figure 1d,e), opening the possibility that WI12*_Rhg1_* may contribute to SCN resistance through the involvement in multiple independent biological pathways. We selected DELLA18 as a top protein interaction candidate for further study and focused on its role in Peking as a result of its colocalization with an SCN resistance QTL in this variety. We did not conclusively demonstrate that DELLA18 is the active or sole gene underlying the QTL, though this is likely. The interactors identified in our screen that were not located in QTL may very well also be involved in the signalling pathway. However, we successfully used QTL co‐localization as a means to prioritize interacting proteins of likely biological importance amongst the many identified using the yeast two‐hybrid system.

**Figure 6:**
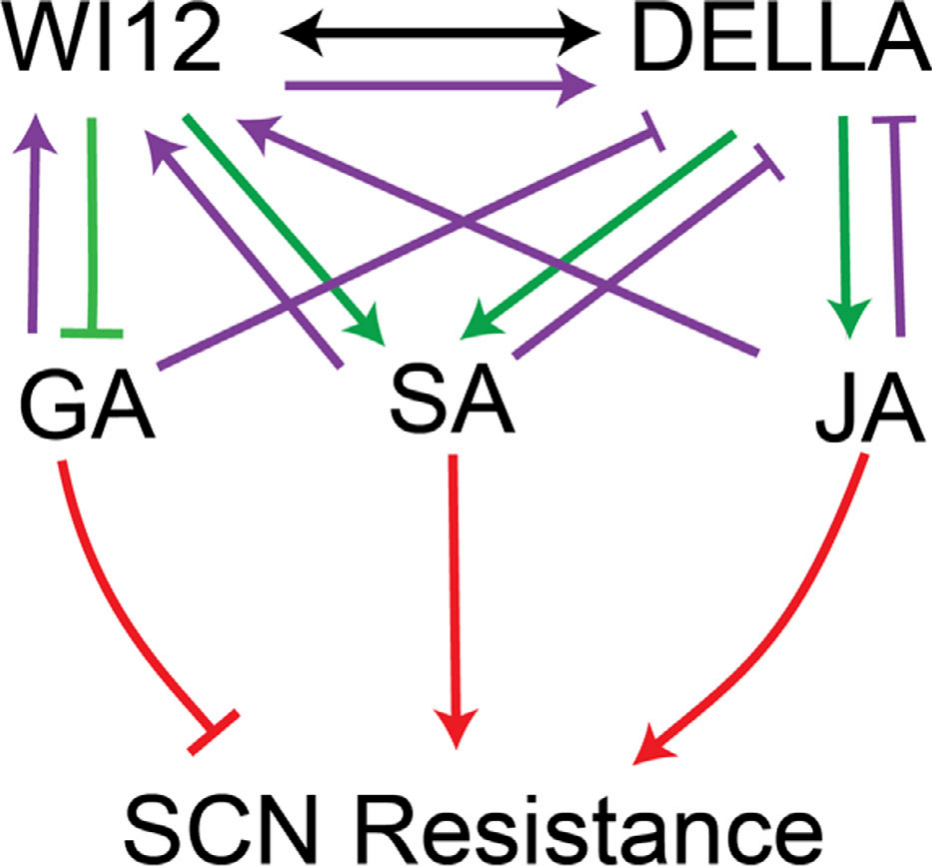
Hormones connect WI12*_Rhg1_* and DELLA in response to nematode infection. The diagram shows interactions supported by evidence in earlier figures and a speculative hierarchy of signalling. The black arrow shows a protein–protein interaction, purple lines an observed influence on transcript expression, green lines an observed influence on hormone levels, and red lines an observed phenotypic effect. Promotion or activation is represented by an arrow and inhibition is represented by an inhibition arrow or arc. Black arrow: WI12*_Rhg1_* and DELLA proteins directly interact. Purple arrows: application of GA, SA and JA induces *WI12_Rhg1_*, but inhibits *DELLA* transcript expression; *DELLA* expression decreases in *WI12_Rhg1_*‐knockout events. Green arrows: knockout of *WI12_Rhg1_* increases the GA_12_ and the GA_53_ content, but decreases the SA, whilst the knockout of *DELLA* decreases the SA and the JA content. Red arrows: application of GA_3_ decreases the SCN resistance, whilst the application of JA or SA increases the SCN resistance.

Importantly, we confirmed that WI12*_Rhg1_* interacts with PDELLA18 *in planta* (Figure 3 and Figure S6) and found that DELLA proteins balance root growth and SCN defence by mediating the interaction between GA and JA pathways, implying that WI12*_Rhg1_* may be involved in DELLA, GA and JA signalling pathways (Figure 6). A previous study demonstrated that *WI12* gene expression is induced by MeJA foliar spray in the halophyte ice plant (Yen *et al*., 2001). In line with the previous studies, we found that *WI12_Rhg1_* gene expression is induced with the treatments of JA, SA or GA and the expression of both *DELLA18* and *GA20ox1* is reduced in WI12*_Rhg1_* knockout roots (Figure 2a,b).

We summarize our observations of signalling‐related interactions in Figure 6. We observed a protein–protein interaction between DELLA and WI12*_Rhg1_*, but we also observe that *DELLA* transcript levels are greatly reduced in the *WI12_Rhg1_* knockout, while *WI12_Rhg1_* transcript levels are similar to unedited hairy roots in the DELLA knockout. Thus, in transcriptional terms, *WI12_Rhg1_* acts upstream of DELLA (Figure 6).

Additionally, we observe that *WI12_Rhg1_* knockout reduces SA levels and *DELLA* knockout reduces both JA and SA levels. Applied JA or SA increases nematode resistance and partially rescues *DELLA* knockout, implying that DELLA acts on nematode resistance at least partly via JA and SA signalling. Applied GA reduces SCN resistance, and as *WI12_Rhg1_* knockout increases the levels of the GAs measured in Figure 2c, we assume that WI12*_Rhg1_* has a repressive effect on overall GA biosynthesis, providing a mechanism for WI12*_Rhg1_* to also act via GA on SCN resistance (Figure 6).

JA, SA and GA all act to increase *WI12 _Rhg1_* transcript levels, and to reduce *DELLA* transcript levels. This provides an interesting feedback response that, along with the effect of *WI12_Rhg1_* knockout on *DELLA*, may imply that *WI12_Rhg1_* is the primary activator of the pathway. Furthermore, we observed that both *DELLA* and *WI12 _Rhg1_* knockout reduce the levels of *GA20ox1* transcript to a similar extent, implying an activating effect on *GA20ox1* transcription for both proteins and a potential mechanistic link to hormone signalling. Increased levels of inactive GA_12_ and GA_53_ were observed in the WI12 *_Rhg1_* knockout lines, also consistent with lower levels of GA20 oxidase activity and/or overall increased GA biosynthesis, but we were not able to observe the same effect in the DELLA knockout lines. We omitted GA20 oxidase and a connection between DELLA and GA from the diagram in Figure 6 because we did not show an effect of *DELLA* knockout on GA levels, but we infer that both WI12*_Rhg1_* and DELLA may act by reducing active GA, likely by reducing GA20 oxidase activity, and that WI12*_Rhg1_* may also exert a global repressive effect on GA biosynthesis, leading to obvious accumulation of inactive GA in the WI12*_Rhg1_* knockout (Figure 2). These results suggest intricate cross‐talk between WI12*_Rhg1_* and DELLA proteins and the GA, SA and JA signalling pathways, which likely mediate at least part of the effect of these proteins on SCN resistance (Figure 6).

### DELLA modulates root growth and nematode defence through hormone pathways

Although plant growth and defence dynamics are known to be modulated by the DELLA, GA and JA network (Hou *et al*., 2010; de Vleesschauwer *et al*., 2016; Yang *et al*., 2012), we were not able to find previous publications regarding the effect of this network on SCN infection. GAs are essential hormones that regulate plant growth and trigger response to environmental stimuli (Hauvermale *et al*., 2012; Salanenka *et al*., 2018). It has been shown that GA can enhance pathogen virulence; for example, GA_3_ foliar treatment promotes nematode infection in rice (Hou *et al*., 2010; Yimer *et al*., 2018). In agreement with the above studies, we found that exogenous application of GA_3_ onto roots significantly increases SCN infection in Peking soybean roots, whilst promoting the growth of longer and thinner first‐order fine roots (Figure 5d). It is likely that this change in root architecture facilitates nematode penetration and infection in roots. On the contrary, JA regulates plant defence against pathogens and is also involved in plant root development (Liechti and Farmer, 2002; Turner *et al*., 2002; Vijayan *et al*., 1998). In line with the previous studies (Hu *et al*., 2017; Nahar *et al*., 2011), we report that exogenous application of MeJA increases the SCN resistance in the Peking unedited hairy roots (Figure 5c), whereas it significantly inhibits root growth (Figure 5a,b). At the same time, the knockout of DELLA decreased SCN resistance and reduced JA and SA content in roots (Figure 4), indicating that the DELLA protein is necessary for both the JA and SA responses to SCN.

DELLA integrates the GA and JA pathways to modulate the dynamics of plant growth and defence (Hou *et al*., 2010; de Vleesschauwer *et al*., 2016; Yang *et al*., 2012). The presence of GA, a growth signal, causes degradation of DELLAs, interrupting the interaction between DELLA and growth‐promoting transcription factors, such as phytochrome interacting factors (PIFs), and liberating PIFs to advance plant growth (Jiang and Fu, 2007; Yang *et al*., 2012; Yoshida *et al*., 2014). We observed that knockout of DELLAs and application of GA both significantly increase the first‐order fine root length, indicating that both the treatments promote the growth of the first‐order fine roots (Figures 4f and 5d). JA signalling causes a defence response to be activated via degradation of JAZ1, which in turn promotes accumulation of DELLA and releases MYC2, causing inhibition of plant growth by the interaction between DELLA and PIFs (Yang *et al*., 2012) and the defence response through the initiation of jasmonate‐defence gene expression by MYC2 (Hou *et al*., 2010, 2013). We found that DELLA is needed for the JA response to SCN, and exogenous application of JA onto DELLA‐knockout roots inhibits root growth and rescues soybean nematode resistance (Figure 5), which is consistent with the expected growth‐defence dynamics of a critical role for DELLA signalling in SCN resistance (Hou *et al*., 2010, 2013). Consistent with our findings, a mutant SLR1, a rice DELLA protein (Ueguchi‐Tanaka *et al*., 2007), has been shown to increase susceptibility to RKN (Yimer *et al*., 2018). Taken together, we conclude that DELLA bridges the GA and JA signalling pathways and modulates root growth and defence against SCN through the hormone signalling pathways.

### The relationship between the SCN resistance, the hormone signalling and the root architecture

Many studies have shown that nematodes affect root growth. When SCN penetrates into roots to form a feeding site, it induces cellular rearrangements and dissolution of root cell walls (Davis and Mitchum, 2005; Williamson and Hussey, 1996), leading to a brown and unhealthy root. Tomato roots are thinner and longer in the presence of bacterial‐feeding nematodes (Mao *et al*., 2007). Whilst the effects of nematodes on root growth are well established, the effect of root architecture on nematode resistance is more challenging to document in a controlled experiment. Prior work has shown that exogenous application of GA_3_ enhances hairy root elongation and lateral root branching (Ohkawa *et al*., 1989). Low concentrations of JA have also been shown to promote lateral root initiation in tomatoes (Tung *et al*., 1996). Our results show that GA_3_ treatment changes root architecture and decreases nematode resistance (Figure 5d,e), whereas JA treatment significantly inhibits root growth, but enhances nematode resistance (Figure 5a,c). We found that knocking out DELLA in Peking hairy roots causes growth of thinner and longer roots, whilst also increasing susceptibility to SCN (Figure 4). The GA_3_ treatment of unedited roots increased the length of the first‐order fine roots, and the extent of nematode infection, compared to untreated controls (Figure 5). Thus, GA and JA both have strong and sensitive effects on root architecture, and also affect SCN resistance. The rapid effect of these hormones on resistance to SCN means that the effect of hormones on nematode infection is unlikely to be mediated solely by morphological effects (Figures 4 and 5); therefore, it is likely that the effects of JA and GA on the plant immunity pathways combine with the root morphology effects to create their observed influence on SCN resistance.

We demonstrate here that the WI12*_Rhg1_* gene interacts with DELLA signalling and hormone systems, which regulate both root architecture and SCN resistance. The above findings implicate the plant hormones GA, JA and SA strongly in SCN resistance. Overall, we have identified an interconnected network of hormones and root architecture controlled by DELLA, which is critical for nematode resistance and is linked to the *Rhg1* locus through an interaction between WI12*_Rhg1_* and DELLA.

## Materials and methods

### Plant and nematode materials

Soybean cultivar Fayette copy number line 99 seedlings (Lee *et al*., 2016) were grown and infected with SCN‐type HG 2.5.7 (purchased from the University of Illinois Plant Clinic) in the greenhouse at the University of Illinois. After 6 days of post‐inoculation, the root samples were collected to create a cDNA library for the yeast two‐hybrid experiment. Soybean cultivars Peking and Essex were obtained from the USDA‐ARS soybean germplasm repository in Urbana, IL and grown in a growth chamber (Conviron, Canada) for hairy root experiments and transcriptional expression profile analysis. HG 2.5.7 eggs were purchased from the University of Illinois Plant Clinic. Plant growth condition, nematode culture and nematode sterilization were performed as previously described (Dong *et al*., 2020).

### Construction of plasmids

To create CRISPR system constructs, gRNAs were designed using CRISPR‐P (Lei *et al*., 2014) and inserted into a Cas9 vector (p201G Cas9; Addgene plasmid #59178) using the Gibson assembly method (Jacobs *et al*., 2015). As DELLA from chromosome 18 (DELLA18) has a high peptide identity with DELLA from chromosome 11 (DELLA11) (Figure S1), two gRNAs were designed to simultaneously target both DELLA18 (D18) and DELLA11 (D11) (Figure S2). The gRNAs and primers are listed in Table S2.

To generate constructs for the BiFC assay, the full‐length cDNA of WI12*_Rhg1_* and Peking‐type DELLA18 (PD18) were cloned into pRZ1152c vector (Dong *et al*., 2020) to generate NmVen210: WI12–PD18: CVen210 using the Gibson assembly method. The full‐length cDNA of WI12*_Rhg1_* and Fayette‐type DELLA18 (FD18) were cloned into the same vector to generate NmVen210: WI12–FD18: CVen210. As a control, WI12*_Rhg1_* was cloned into the same vector to generate a parent vector NmVen210: WI12–X: CVen210. The primers are listed in Table S1.

To build plasmids for both FRET acceptor photobleaching analysis and the subcellular localization, the full‐length cDNA of PD18 was cloned into a modified pSM101 vector (Mankin and Thompson, 2001; Melito *et al*., 2010) fused with CFP using the Gibson assembly method to generate the pSM101‐CFP‐PD18 construct. WI12*_Rhg1_* was cloned into the same vector fused with YFP to generate the pSM101‐YFP‐WI12 construct. The primers are listed in Table S1.

### Yeast two‐hybrid assay and analysis of the location of candidate genes

Yeast two‐hybrid library was constructed using the Matchmaker^®^ Gold Yeast Two‐Hybrid System and Make Your Own ‘Mate & Plate’ Library system manual (Clontech, San Jose, CA, USA). The procedure used for screening of the cDNA library and identification of candidate proteins can be found in our previous study (Dong *et al*., 2020).

A cDNA library from SCN‐infected Fayette 99 root was constructed with sizes ranging from approximately 0.5 to 10 kilobase pairs (Figure S3). The yeast two‐hybrid library was screened using progressively higher stringency, from medium strength selection on TDO plates to the highest stringency QDO plates, at three different time stages (3, 5 and 7 days after library transformation) (Figure S4). The surviving colonies from QDO plates were amplified using yeast colony PCR followed by Sanger sequencing to identify the candidate genes, which ranged in size from approximately 0.5 to 1.5 kilobase pairs (Figure S5).

To confirm the interactions between candidate proteins and WI12*_Rhg1_*, we employed a plate assay (Dong *et al*., 2020) and α‐Galactosidase assay (Yeast Protocols Handbook, Clontech). The full‐length cDNAs of FD18 and PD18 were individually cloned into the prey vector to confirm the interactions with the bait vector harbouring WI12*_Rhg1_*. The association of the physical location of the candidate genes with the previously identified SCN QTL was analyzed as described in Dong *et al*. (2020).

### Transient expression assays

All the constructs were transformed into *Agrobacterium rhizogenes* strain K599 strain by the freeze‐thaw method (Jyothishwaran *et al*., 2007) and into *Agrobacterium tumefaciens* strain GV3101 using the electroporation method (Nagel *et al*., 1990). *A*. *tumefaciens* strain GV3101 and *A*. *rhizogenes* strain K599 was used for the transient infection of *Nicotiana benthamiana* leaves (Gookin and Assmann, 2014) and wounded soybean cotyledons to generate soybean hairy roots (Chen *et al*., 2018; Cook *et al*., 2012), respectively.

### Mutant screening, SCN demographics assays and measurement of root morphology

Independent hairy roots were collected from different cotyledons and individually cultured on the ICM (co‐cultivation culture medium) plates (Chen *et al*., 2018; Cook *et al*., 2012). After growth in the dark at room temperature for 1 week, a 2‐cm root tip from the independent hairy root was collected and placed onto a new ICM plate. The remaining part of the root was collected for genomic DNA extraction (Murray and Thompson, 1980). *WI12_Rhg1_*, *D18* and *D11* were amplified using primers shown in Table S2 and Figure S2. The PCR amplicons were cleaned up using dNTP with Exonuclease I (NEB) and Shrimp Alkaline Phosphatase (NEB) followed by Sanger sequencing. gRNA editing efficiencies were determined using the inference of CRISPR editing (ICE) analysis (Synthego, Menlo Park, CA) (Hsiau *et al*., 2018). If any editing was detected in the root based on the ICE analysis, the original 2‐cm root tip, which had been moved to a fresh ICM plate, was used for a nematode demographics assay with 400 J2 applying onto roots. After 12‐days post‐inoculation (dpi), the root was stained using the acid fuchsin method (Bybd *et al*., 1983) and nematode development in the whole root was counted (Cook *et al*., 2012; Dong *et al*., 2020). Independent transgenic events were generated from different cotyledons. The total number of independent‐edited transgenic hairy roots from at least three independent experiments is shown in Figures 1c and 4c. For DELLA knockout roots and control roots, the length of the first‐order fine roots and the diameters of the first‐order fine roots and main root were measured from at least 10 randomly selected roots. Measurements were performed using Adobe Illustrator (Adobe, San Jose, CA). The unbranched root segments that end in root tips are classified as the first‐order fine roots (Pregitzer *et al*., 2002).

### BiFC assay

NmVen210:WI12–PD18:CVen210, NmVen210:WI12–FD18:CVen210 or NmVen210:WI12–X:CVen210 were individually transformed into both GV3101 and K599 strains which were then used for transient infection of both *N*. *benthamiana* leaves and Peking cotyledons, respectively, as described above. Tobacco leaf cells were imaged under a 40× objective with water‐immersion after 48 h infiltration and 1 mm root tips were observed under a 63× objective with oil‐immersion using a laser‐scanning microscope LSM 880 (Carle Zeiss Inc., Thornwood, NY). Imaging was performed and fluorescent signals were detected using an excitation wavelength of 514 nm and an emission wavelength of between 516 and 565 nm. *Agrobacterium* transfection and fluorescence observation were performed in *N*. *benthamiana* and soybean hairy root as described previously (Dong *et al*., 2020; Gookin and Assmann, 2014).

### Subcellular localization of WI12*_Rhg1_* and DELLA18 in *N*. *benthamiana* and Peking root

pSM101‐YFP, pSM101‐CFP, pSM101‐YFP‐WI12, pSM101‐CFP‐DELLA18, nuclear localization sequences pSM101‐YFP‐41V (Dong *et al*., 2020; Lange *et al*., 2007) and membrane‐targeting sequences pSM101‐YFP‐51V (Batistič *et al*., 2008; Dong *et al*., 2020) were individually transformed into both GV3101 and K599 strains which are used for infection of *N*. *benthamiana* leaves and soybean roots, respectively. Tobacco leaf and root cells were observed and imaged using the LSM 880 under a 40× and a 63× objective, respectively as above. CFP was excited at 458 nm and the emission signal was collected at 481 nm, whilst YFP was excited at 514 nm and the emission wavelength was 544 nm.

### FRET acceptor photobleaching assay

Two *A*. *rhizogenes* K599 strains, each carries one different plasmid (i.e. one plasmid with a protein fused to CFP and another plasmid with another protein fused to YFP), were mixed in a 1 : 1 ratio and used to infect Peking cotyledons to generate hairy roots. There are four strain pairs: pSM101‐CFP and pSM101‐YFP (background control, CFP‐YFP); pSM101‐YFP‐WI12 and pSM101‐CFP‐PD18 (sample, WI‐DELLA); pSM101‐YFP‐WI12 and pSM101‐CFP (control, WI‐CFP); pSM101‐CFP‐DELLA18 and pSM101‐YFP (control, DELLA‐YFP).

Peking root tips were examined under a 63× objective with the LSM 880. The YFP (acceptor) and CFP (donor) fluorescence were excited using 514 and 458 nm excitation argon lasers, respectively. The acceptor YFP fluorescence in the region of interest was bleached using a 514 nm argon laser line. The FRET efficiency was calculated using the following formula: FRET efficiency = (I_6_–I_5_)/I_6_ × 100 (Tunc‐Ozdemir *et al*., 2016). The photobleaching and image acquisitions were performed and analyzed as described previously (Karpova and McNally, 2006; Schindelin *et al*., 2012, 2015; Tunc‐Ozdemir *et al*., 2016).

### Hormone content measurements

Two centimetres of DELLA‐knockout, WI12*_Rhg1_*‐knockout or unedited roots (hairy roots carrying pCas9 vector without any gRNAs) derived from Peking and Essex soybean genotypes, were grown on ICM plates for 12 days, then 100 mg of fresh roots were sampled, quickly frozen in liquid nitrogen and stored at −80°C. The hormone measurements were performed by the Creative Proteomics Company (Shirley, NY). Hormones were extracted from the root samples (100 mg) using cold methanol:acetonitrile (50 : 50, v/v) spiked with deuterium‐labelled internal standards (mixture of D2‐JA, D4‐SA, D2‐GA_53_, D2‐GA_12_). The tissue samples were disrupted using the TissueLyserII (Qiagen). After centrifugation at 16 000 ***g***, the supernatants were collected, and extraction of the pellet was repeated one more time. The supernatants were pooled and dried down using a speed‐vac. The pellets were re‐dissolved in 200 μL of 15% methanol. The gradient of the mobile phases A (0.1% acetic acid) and B (0.1% acetic acid/90% acetonitrile) was as follows: 5% B for 1 min, to 60% B in 4 min, to 100% B in 2 min, hold at 100% B for 3 min, to 5% B in 0.5 min. The flow rate was 0.45 mL/min. The hormones were detected using MRM transitions that were optimized using standards. The instrument was set up to acquire positive and negative ion switching. For quantification, an external standard curve was prepared using a series of standard samples containing different concentrations of unlabeled hormones and fixed concentrations of the deuterium‐labelled standards mixture.

### Hormone treatments for SCN demographics assays

A solution of GA, MeJA or SA was added uniformly onto the surface of ICM plates using sterile glass beads. Two‐centimetre roots were then added and grown on each plate. After 1 day of growth on the plate, each root was treated with 400 juvenile HG 2.5.7‐type nematodes. For GA_3_ treatments, Peking unedited roots were treated with four different concentrations of GA_3_ (0, 0.1, 1 and 10 μm). One day later, 400 J2 HG 2.5.7‐type nematodes were added around the roots. An image of the whole root was captured 12 days after inoculation using a Leica MZ16 stereomicroscope with an attached camera. The length of first‐order fine roots and the diameters of the first‐order fine roots and main root were measured from at least 10 randomly selected roots. Measurements were performed using Adobe Illustrator. After imaging, the whole root was stained, and nematode development was quantified as described above. For MeJA treatment, DELLA‐edited roots and unedited roots both from Peking were placed on ICM plates with two MeJA concentration treatments (0, 150 μm) and inoculated with HG 2.5.7‐type SCN followed by a nematode demographics assay and the root architecture measurement as described above. For SA treatments, DELLA‐edited roots were placed on ICM plates with 1 mm SA and inoculated with HG 2.5.7‐type SCN followed by nematode demographics assays as described above.

### Exogenous hormonal application for RT‐qPCR

A 2‐cm Peking unedited hairy root was transferred to a fresh ICM agar plate and grown for 7 days. Then, 150 μl of either 10 μm GA_3_, 150 μm MeJA, 1 mm SA or water as control were applied onto the whole roots. Root samples were collected at 0 and 4 h for RT‐qPCR.

### Total RNA extraction and RT‐qPCR

Leaf, stem and root samples from Peking and Essex seedlings (Chang *et al*., 1993), DELLA‐knockout roots, WI12*_Rhg1_*‐knockout roots, and GA_3_, SA and MeJA treatments of roots were collected for RNA extraction. First‐strand cDNA was synthesized from root mRNA using the High Capacity cDNA Reverse Transcription Kit (ThermoFisher Scientific, Waltham, MA). Quantitative PCR (qPCR) was performed by amplifying cDNAs using the Power SYBR^®^ Green PCR Master Mix (ThermoFisher Scientific) in a LightCycler 480 instrument (Roche, Indianapolis, IN). The relative expression values shown in the figures were determined using ubiquitin *(Glyma.20G141600)* as a reference gene and followed the ΔCt measurement method (Livak and Schmittgen, 2001). Five biological replicates with four technical repeats each were performed for each analysis. Primers used for qPCR are listed in Table S1.

### Statistical analysis

All the *P*‐values provided were generated using the two‐tailed Welch’s *t*‐test and the analysis was performed via the IBM SPSS software package (Armonk, NY).

## Conflict of interest

The authors declare no competing interests.

## Author contributions

J.D. and M.E.H. together developed the experimental plan, whilst J.D. performed the experimental procedures. J.D. and M.E.H. together performed the data analysis and interpretation. J.D. and M.E.H. wrote, edited and revised this manuscript.

## Supporting information


**Figure S1** DELLA proteins domain view and alignment of two peptide sequences between DELLA18 and DELLA11.
**Figure S2** CRISPR‐Cas9 with gRNA construction and diagram of gene, gRNAs and primers.
**Figure S3** cDNA library constructed from SCN‐infected Fayette root RNA.
**Figure S4** Sequential transformation of cDNA library into bait strain carrying WI12*_Rhg1_* protein.
**Figure S5** Confirmation recombinants in cDNA library by yeast colony PCR.
**Figure S6** BiFC assays were used to demonstrate the interaction between WI12*_Rhg1_* and DELLA18 *in planta*.
**Figure S7** Subcellular localization of WI12*_Rhg1_* and PDELLA18 proteins in *N. benthamiana*.
**Figure S8** Subcellular localization of WI12*_Rhg1_* and PDELLA18 proteins soybean roots.
**Figure S9** Investigation of protein interactions in Peking hairy root using FRET acceptor photobleaching method.
**Figure S10** Comparison of the sequences between W82, Essex, Fayette 99 and Peking.
**Figure S11** CRISPR gRNA editing efficiencies for guides targeting DELLA18 and its homeolog DELLA11 in Peking (black) and Essex (grey).
**Table S1** Primers used in this study.
**Table S2** Primers used for CRISPR‐Cas9 genome editing system.
**Table S3** Correlation between DELLAs and QTL.

**Table S4** Candidate proteins interacted with WI12*_Rhg1_*.
