# Supplementary material for: WI12 Rhg1 interacts with DELLAs and mediates soybean cyst nematode resistance through hormone pathways
Source: Plant Biotechnol J. 2021 Oct 5;20(2):283–96. doi: 10.1111/pbi.13709 (PMC8753364; doi:10.1111/pbi.13709)
Supplement: Supplementary file 1 — Figure S1 DELLA proteins domain view and alignment of two peptide sequences between DELLA18 and DELLA11. Figure S2 CRISPR‐Cas9 with gRNA construction and diagram of gene, gRNAs and primers. Figure S3 cDNA library constructed from SCN‐infected Fayette root RNA. Figure S4 Sequential transformation of cDNA library into bait strain carrying WI12 Rhg1 protein. Figure S5 Confirmation recombinants in cDNA library by yeast colony PCR. Figure S6 BiFC assays were used to demonstrate the interaction between WI12 Rhg1 and DELLA18 in planta. Figure S7 Subcellular localization of WI12 Rhg1 and PDELLA18 proteins in N. benthamiana. Figure S8 Subcellular localization of WI12 Rhg1 and PDELLA18 proteins soybean roots. Figure S9 Investigation of protein interactions in Peking hairy root using FRET acceptor photobleaching method. Figure S10 Comparison of the sequences between W82, Essex, Fayette 99 and Peking. Figure S11 CRISPR gRNA editing efficiencies for guides targeting DELLA18 and its homeolog DELLA11 in Peking (black) and Essex (grey). Table S1 Primers used in this study. Table S2 Primers used for CRISPR‐Cas9 genome editing system. Table S3 Correlation between DELLAs and QTL. [file PBI-20-283-s002.pdf]

Supporting information

"WI12$_{Rhg1}$ interacts with DELLAs and mediates soybean cyst nematode resistance through hormone pathways"

Jia Dong[1], Matthew E. Hudson[1,*]

1 University of Illinois Urbana-Champaign, Department of Crop Sciences

[*] Corresponding author: mhudson@illinois.edu

Supporting Figs

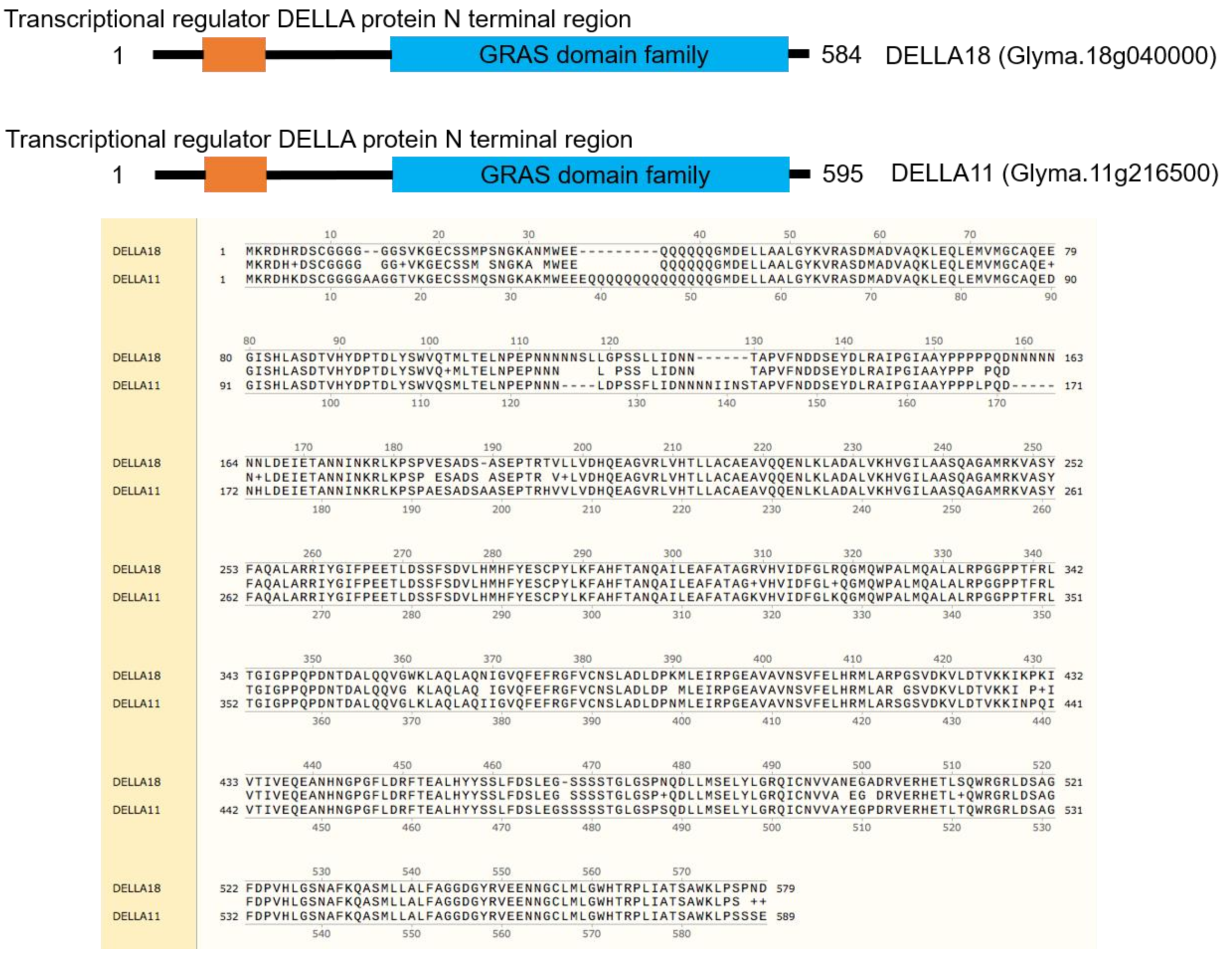

Figure S1. DELLA proteins domain view and alignment of two peptide sequences between DELLA18 and DELLA11.

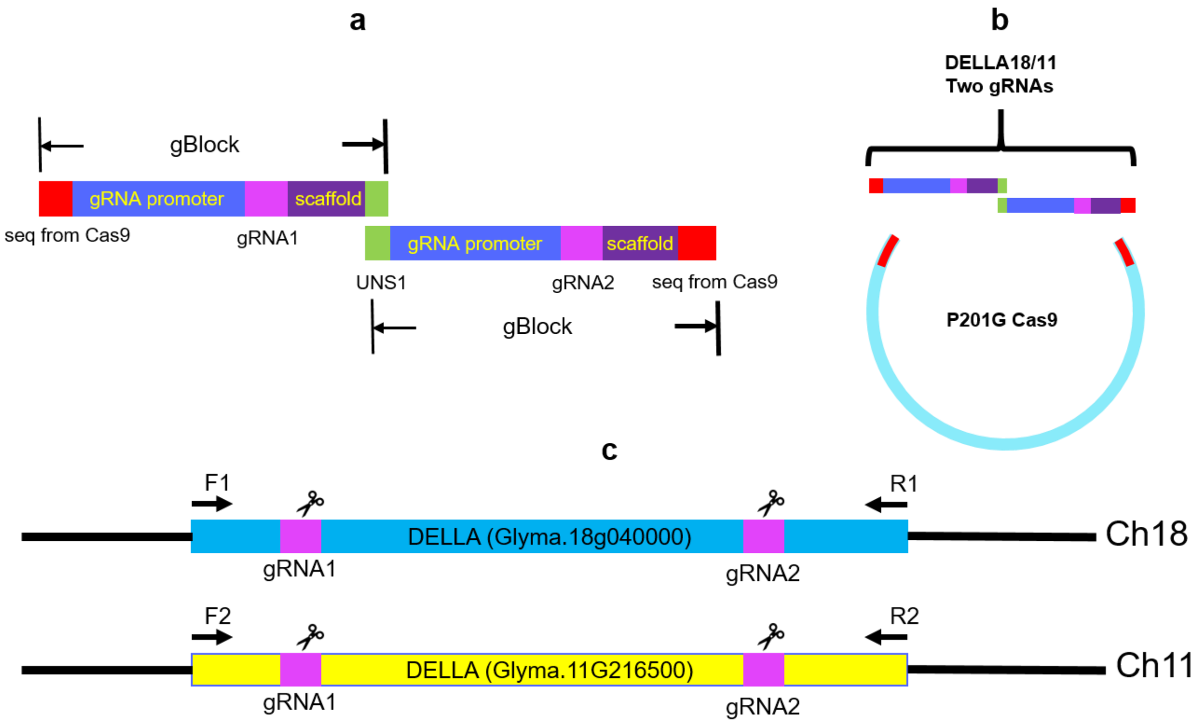

Figure S2. CRISPR-Cas9 with gRNA construction and diagram of gene, gRNAs, and primers. a, gBlock diagram. Each gBlock contains sequences from Cas9, gRNA promoter, gRNA target sequence, and gRNA scaffold. Separate gBlocks contain overlapping homologous sequences for Gibson Assembly reactions. For Cas9-gRNA construct targeting two DELLA protein, two gBlocks were used: gBlock1[18-11] containing gRNA1 targeting DELLA18 and DELLA11, gBlock2[18-11] containing gRNA2 targeting DELLA18 and DELLA11. **b,** Cas9-gRNA construct. Construct targeting two DELLA genes was constructed from two gBlocks: gBlock1[18-11], gBlock2[18-11]. **c,** Diagram of DELLAs, gRNAs, and primers. Each DELLA is targeted by two gRNAs and each primer is specific to only one DELLA. After PCR amplification, the PCR product is sequenced using the forward and reverse primers to determine gRNA1 and gRNA2 editing efficiency, respectively.

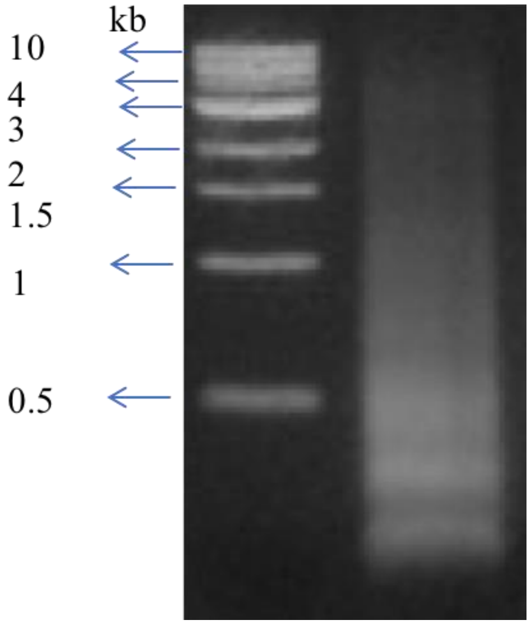

Figure S3. cDNA library constructed from SCN-infected Fayette root RNA. The size range of cDNA was from less than 500 bp to 10 kb.

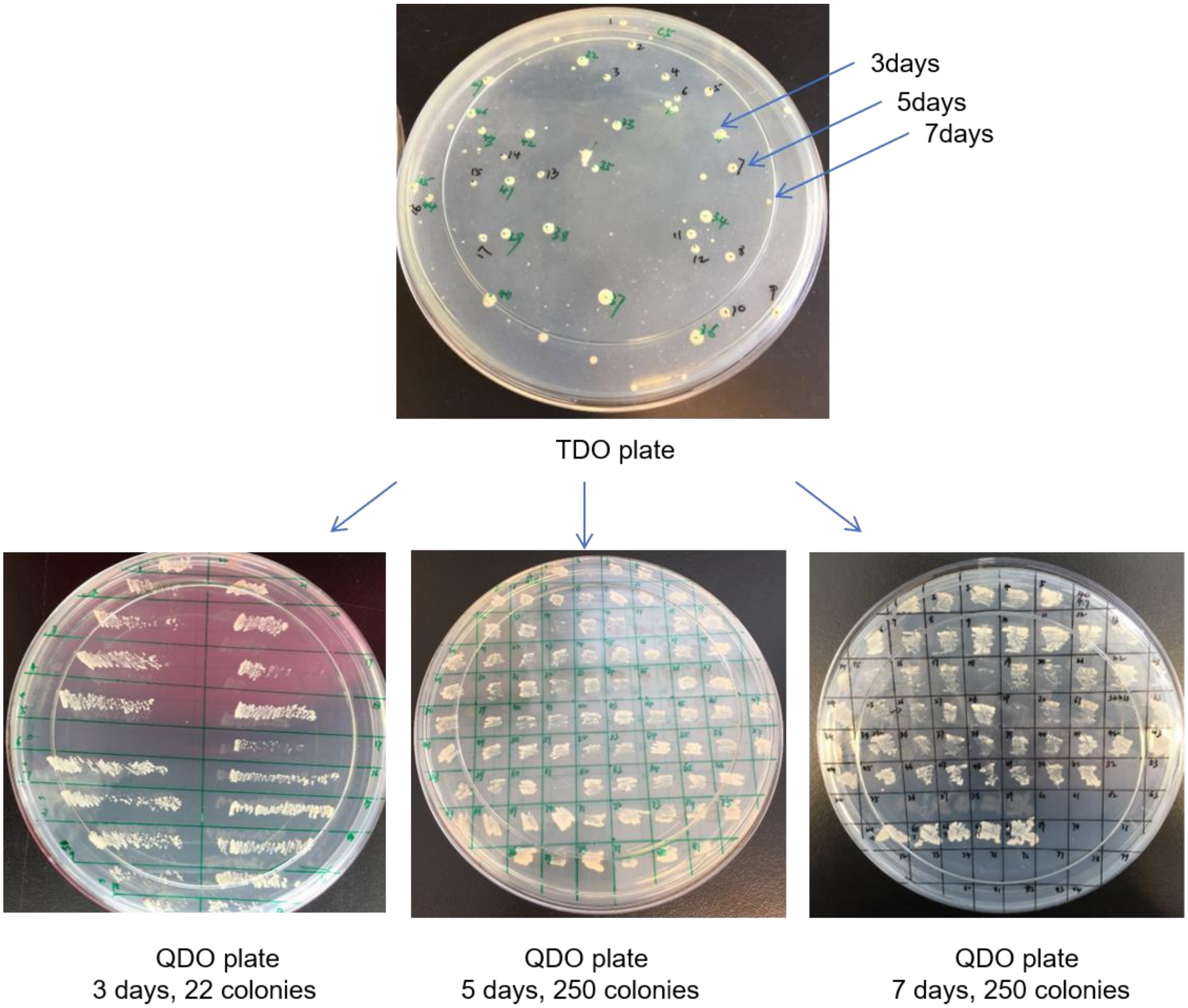

Figure S4. Sequential transformation of cDNA library into bait strain carrying WI12$_{Rhg1}$ protein. Restreak the colonies from TDO plate onto higher stringent selection plate QDO plate after 3, 5, 7 days cDNA library transformation. TDO plate: Triple drop out of tryptophan, leucine and histidine plate; QDO plate: quadruple drop out of tryptophan, leucine, histidine and adenine plate.

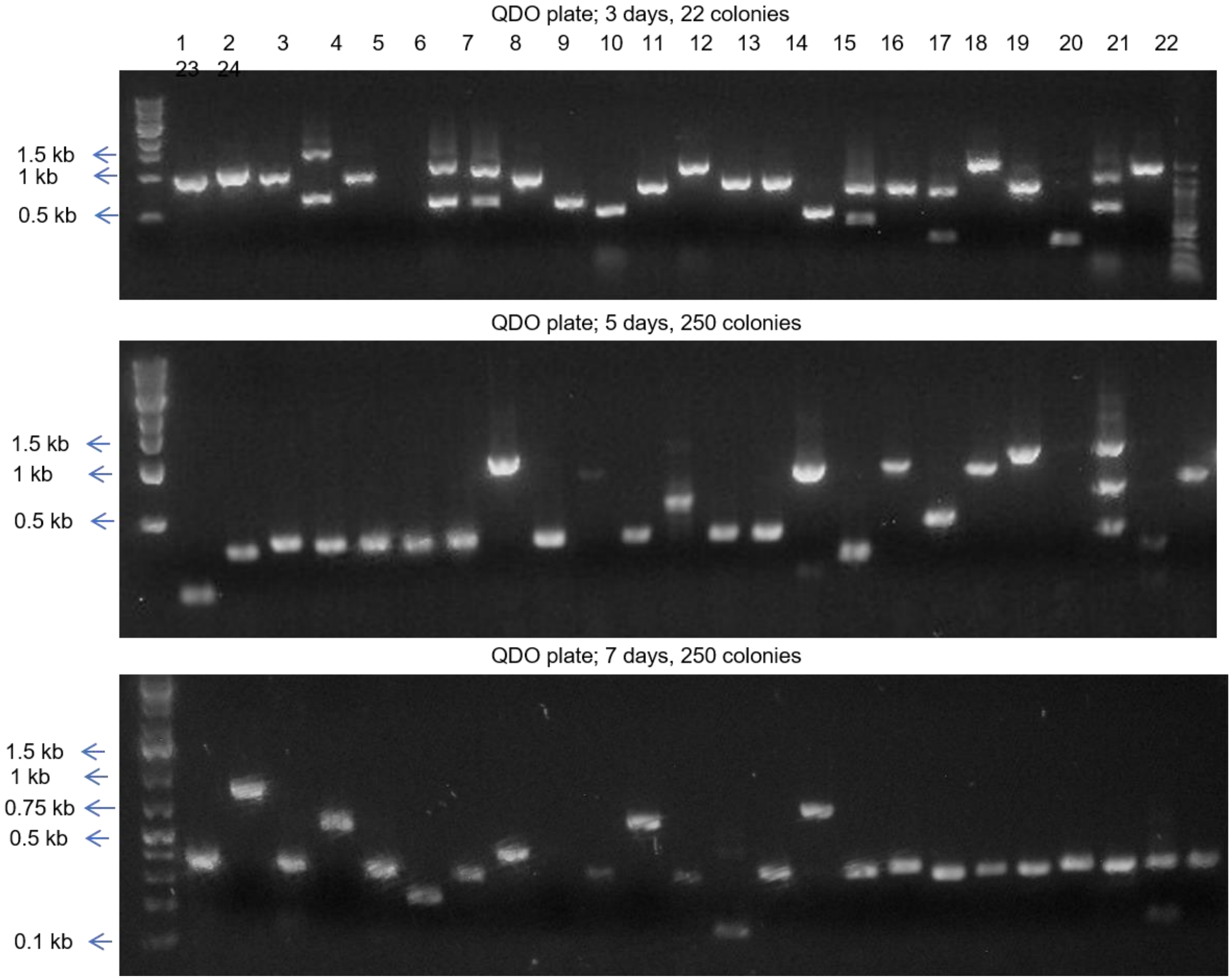

Figure S5. Confirmation recombinants in cDNA library by yeast colony PCR.
The size range of recombinants is from ~150 bp to 1.5 kb.

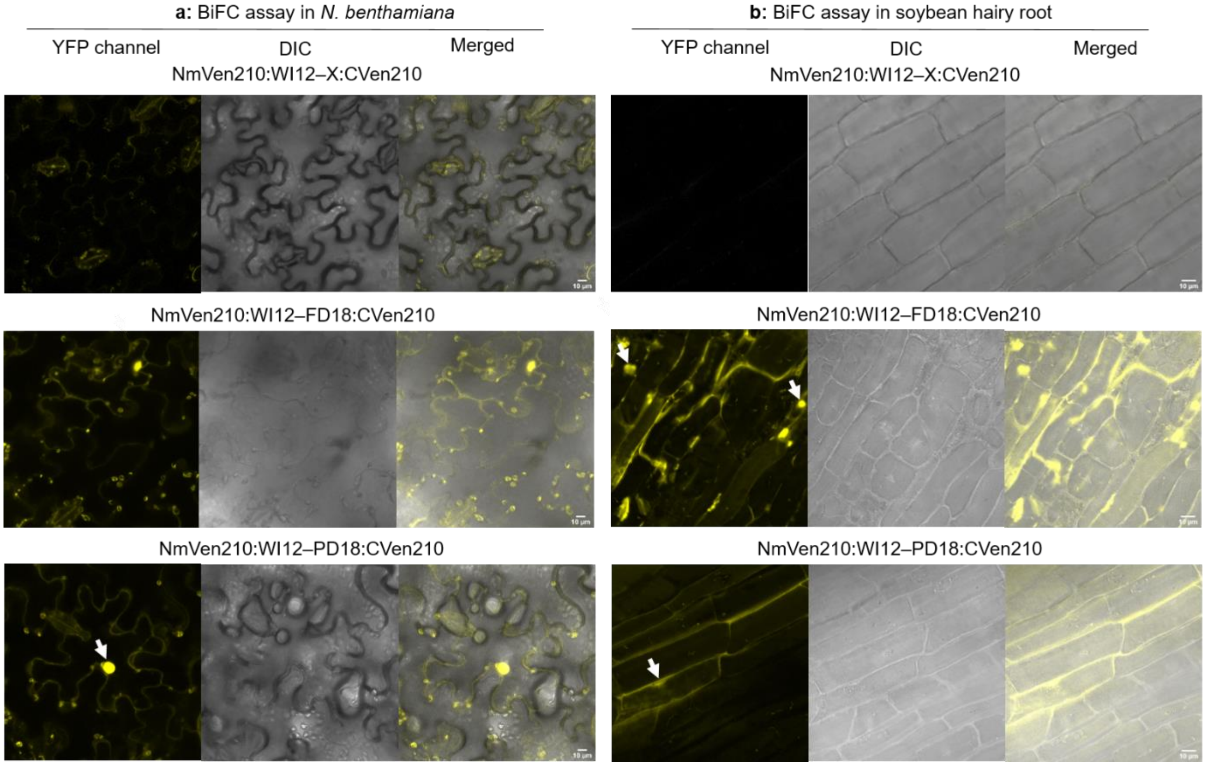

Figure S6. BiFC assays were used to demonstrate the interaction between WI12$_{Rhg1}$ and DELLA18 *in planta*. **a,** *N. benthamiana* epidermal cells and **b,** soybean hairy root cells. (Top) Parent vector NmVen210:WI12–X:CVen210 shows the weak background signal. (Middle) Increased BiFC fluorescent signal compared to control in both *N. benthamiana* epidermal and Fayette 99 soybean hairy root cells holding plasmid NmVen210:WI12–FDELLA18:CVen210 indicates interaction occurred between WI12$_{Rhg1}$ (WI12) and FDELLA18 (FD18). (Bottom) Increased BiFC fluorescent signal compared to control in both *N. benthamiana* epidermal and Peking soybean hairy root cells holding plasmid NmVen210:WI12–PDELLA18:CVen210 indicates interaction between WI12$_{Rhg1}$ (WI12) and PDELLA18 (PD18). Each BiFC sample is represented by three images: (Left) fluorescent image obtained from YFP channel, (Middle) a DIC (differential interference contrast) image, and (Right) a merged image from YFP channel and DIC. The nucleus is labeled with a white arrow. Scale bar = 10 μm.

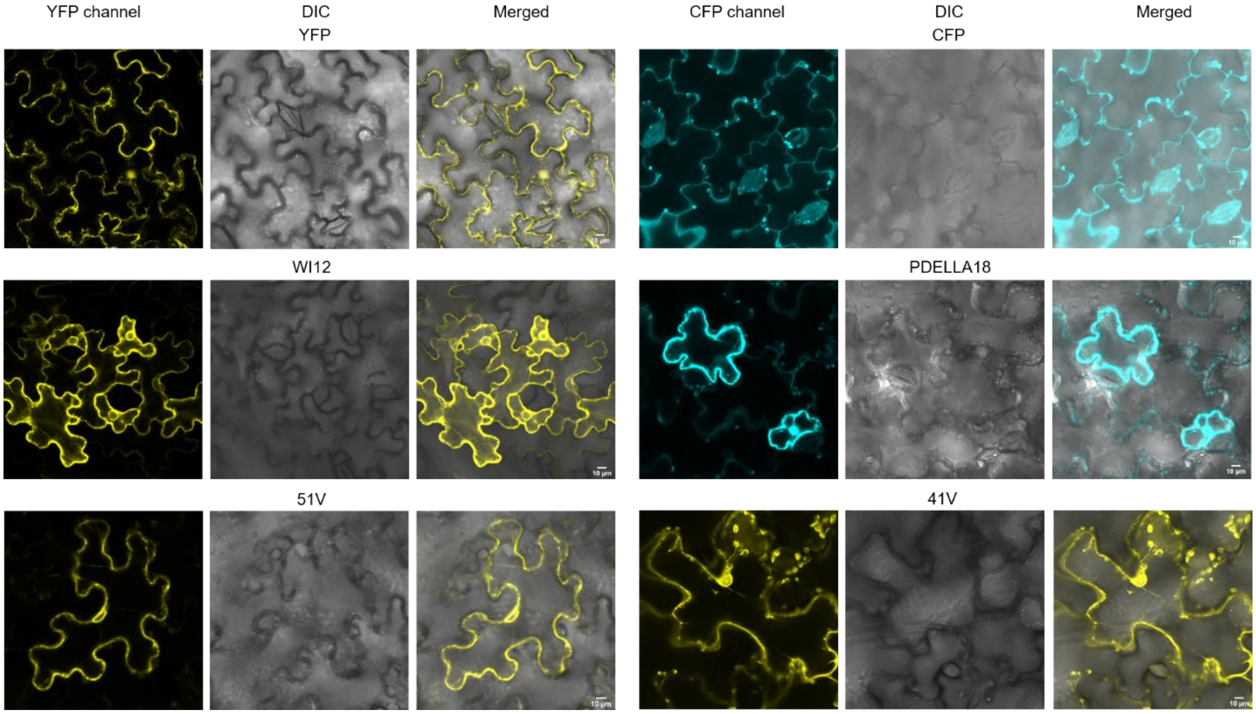

Figure S7. Subcellular localization of WI12$_{Rhg1}$ and PDELLA18 proteins in *N. benthamiana.* Plasmids expressed in *N. benthamiana* epidermal cells. YFP: pSM101-YFP, CFP: pSM101-CFP, WI12: pSM101-YFP-WI12, PDELLA18: pSM101-CFP-PDELLA18, 51V: pSM101-YFP-51V (membrane-targeted YFP), and 41V: pSM101-YFP-41V (nuclear-targeted YFP). Each sample is represented by three images: (Left) fluorescent image obtained from YFP or CFP channel, (Middle) a DIC (differential interference contrast) image, and (Right) a merged image from YFP or CFP channel and DIC. Scale bar = 10 µm.

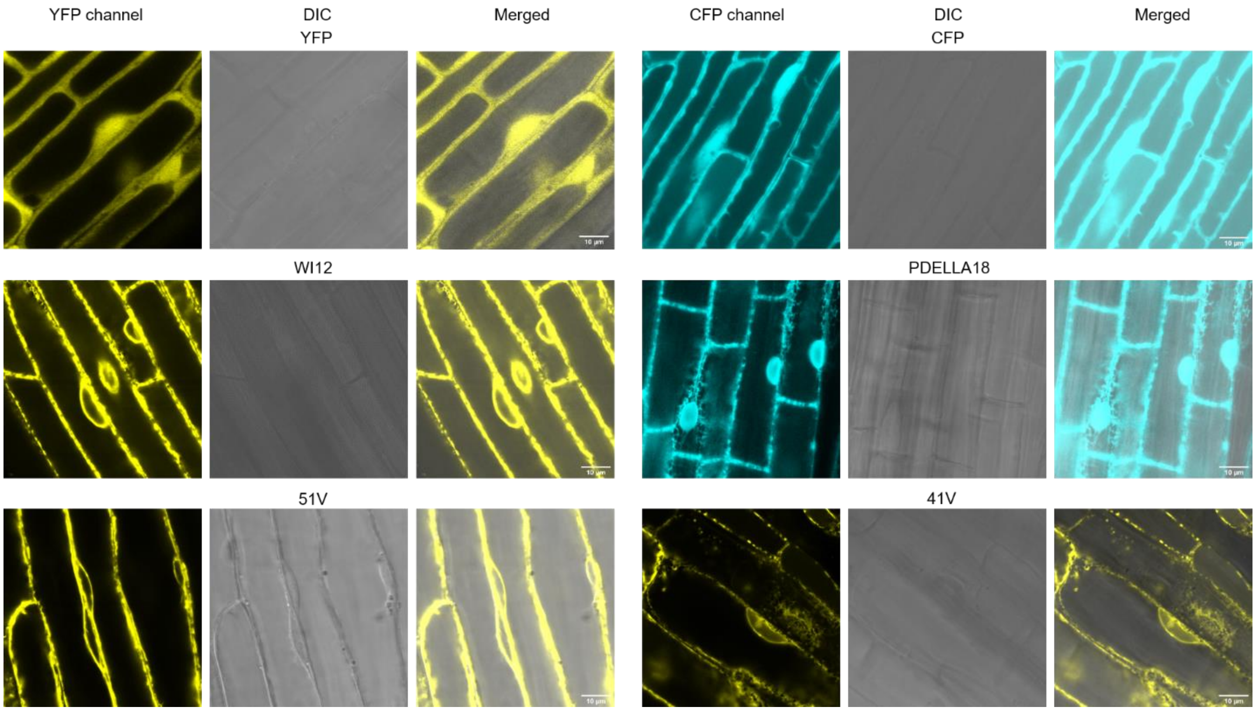

Figure S8. Subcellular localization of WI12$_{Rhg1}$ and PDELLA18 proteins soybean roots. Plasmids expressed in Peking hairy root cells. YFP: pSM101-YFP, CFP: pSM101-CFP, WI12: pSM101-YFP-WI12, and PDELLA18: pSM101-CFP-PDELLA18, 51V: pSM101-YFP-51V (membrane-targeted YFP), and 41V: pSM101-YFP-41V (nuclear-targeted YFP). Each sample is represented by three images: (Left) fluorescent image obtained from YFP or CFP channel, (Middle) a DIC (differential interference contrast) image, and (Right) a merged image from YFP or CFP channel and DIC. Scale bar = 10 μm.

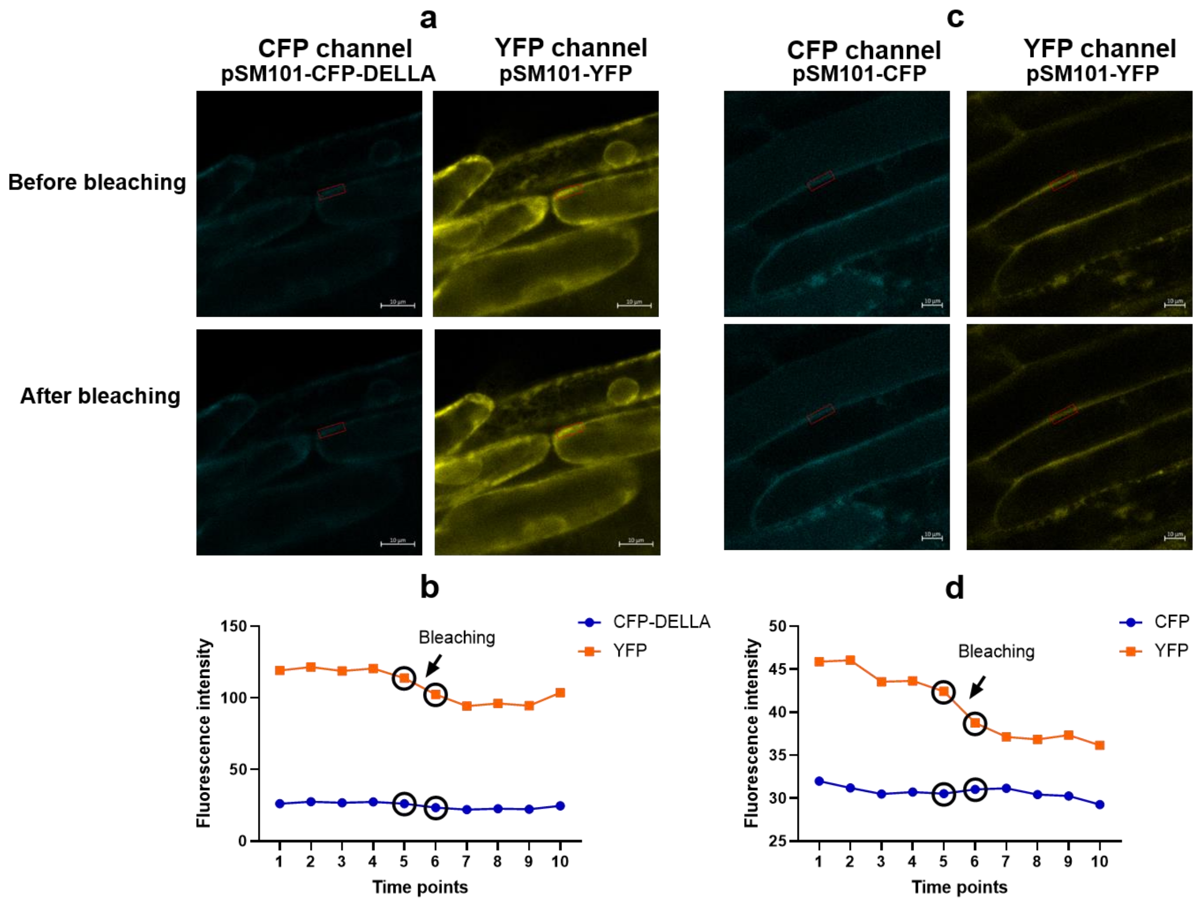

Figure S9. Investigation of protein interactions in Peking hairy root using FRET acceptor photobleaching method. a, Fluorescent images show the fluorescence brightness changes in CFP (pSM101-CFP-PDELLA18) and YFP (pSM101-YFP) channels between the 5th time point (right before bleaching) and the 6th time point (right after bleaching). b, Quantification of effects of photobleaching on CFP (pSM101-CFP-PDELLA18) and YFP (pSM101-YFP) fluorescence intensity. A decrease in YFP fluorescence was observed during photobleaching without a corresponding increase in CFP fluorescence. c, Fluorescent images show the fluorescence brightness changes in CFP (pSM101-CFP) and YFP (pSM101-YFP) channels between the 5th time point (right before bleaching) and the 6th time point (right after bleaching). d, Quantification of effects of photobleaching on CFP (pSM101-CFP) and YFP (pSM101-YFP) fluorescence intensity. A decrease in YFP fluorescence was observed during photobleaching without a corresponding increase in CFP fluorescence. Scale bar = 10 μm.

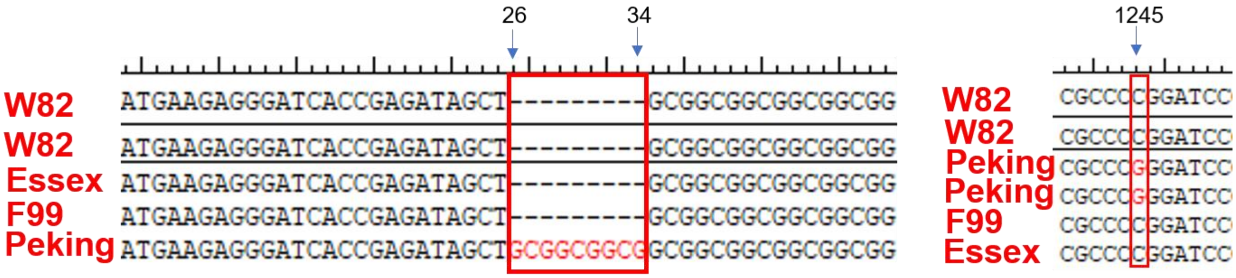

Figure S10. Comparison the sequences between W82, Essex, Fayette 99 and Peking. Peking have 9 bps insertion and 1 SNP (nonsense mutation) compare with W82, Essex and Fayette 99.

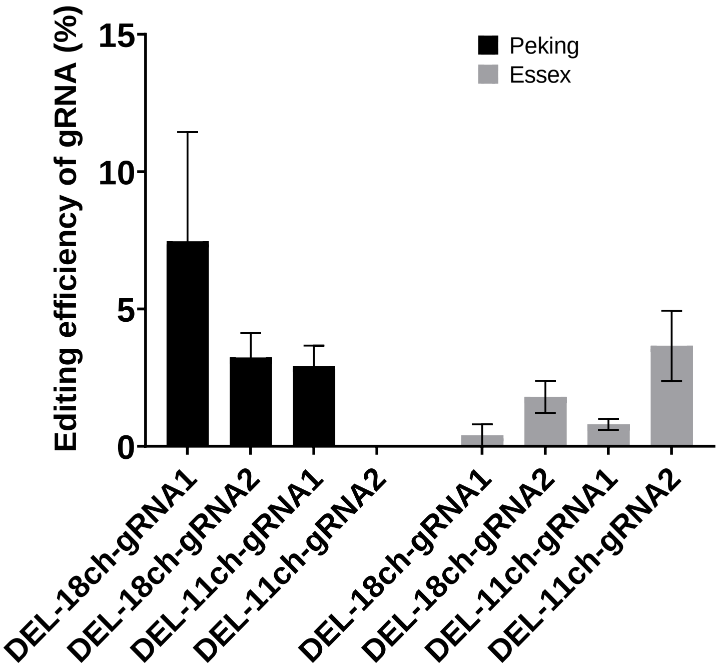

Figure S11. CRISPR gRNA editing efficiencies for guides targeting DELLA18 and its homeolog DELLA11 in Peking (black) and Essex (grey).

Supporting Tables

Table S1. Primers used in this study.

Description	Gene ID	Forward/ Reverse 5' to 3'
Gene cloning	Glyma.18g022700	F: GTACGTACCATATGATGCGCATGCTCACCGG
		R: GTACGTACCCCGGGTTATATTGCGAGAACCAAACCGG
cDNA library amplification	5' cDNA PCR	F: TTCCACCCAAGCAGTGGTATCAACGCAGAGTGG
	3' cDNA PCR	R: GTATCGATGCCCACCCTCTAGAGGCCGAGGCGGCCGACA
Sequencing inserts in the AD/library plasmids	T7 Primer	F: TAATACGACTCACTATAGGGCG
	3' DNA-BD Primer	R: AGATGGTGCACGATGCACAG
RT-qPCR	Glyma.18g022700	F: CTTTCTCGAACTGGCTAACTCTCAG
		R: GATCATCCCATCAGTGACGGTC
	Glyma.18G040000	F: TTGAATCAGCAGACTCCGCG
		R: GCTTCAGATTCTCCTGCTGG
	Ubiquitin gene	F: GTGTAATGTTGGATGTGTTCCC
		R: ACACAATTGAGTTCAACACAAACCG
	Glyma.09G149200	F: AATGTCTCAACCACAAAAGCACCACCAAGAG
		R: GAACCCCCCCAAGTCAATGAGAGGGACAC
Subcellular localization and FRET	Glyma.18g022700	F: GCATGGACGAGCTGTACAAGATGCGCATGCTCACCGG
		R: CTGGGGACCTGGGTACCTTATATTGCGAGAACCAAACCGG
	Glyma.18G040000	F: GCATGGACGAGCTGTACAAGATGAAGAGGGATCACCGA
		R: CTGGGGACCTGGGTACCCTACAGTTTACAGTGCAAGTCG
BiFC	Glyma.18g022700	F: TCCATGCATGGGGCGCGCCCTAGGATGCGCATGCTCACCGG
		R: CCCGGGTCTAGATTAACTAGTTTTATATTGCGAGAACCAAACCGG
	Glyma.18G040000	F: TACAATTACATTTACAATTACCATGAAGAGGGATCACCGAG
		R: CACGTGACTACGTAGGGACCCCATCAGTTTACAGTGCAAGTCGTTC

Table S2. Primers used for CRISPR-Cas9 genome editing system.

Knockout events	Gene ID	gRNA sequence	Forward/ Reverse primers
Double knockout for DELLA from chromosome 18 and 11	Glyma.18g040000	GGAATCGTCGTTGAAAACAG	F:CTTTCCTATGACCCTATTCCAAATC
		GAGCAGTTGGAGATGGTCAT	R: CGCGGAGTCTGCTGATTCAA
	Glyma.11G216500	GGAATCGTCGTTGAAAACAG	F: TTTTCCTATGACCCAATTCCAAATC
		GAGCAGTTGGAGATGGTCAT	R: GGCGGAGTCTGCTGATTCCG
Single knockout of Glyma.18g022700	Glyma.18g022700	CGATGGACTGCGGAACGAAT	F: CTTTCTCGAACTGGCTAACTCTCAG
			R: GATCATCCCATCAGTGACGGTC

Table S3. Correlation between DELLAs and QTL.

DELLA Proteins	Locate in QTL	Parent lines	Literature
DELLA18	SCN 44-3	Williams 82 × PI 437655	[1]
	1cM away from SCN 8-1	Essex × Forrest	[2]
	Lateral root density 1-1	Essex × Forrest	[3]
DELLA11	SCN 39-2	Essex × PI 437654	[4]

Table S4. Candidate proteins interacted with WI12$_{\mathit{Rhg1}}$.

Reference

1. Jiao, Y. *et al.* Identification and evaluation of quantitative trait loci underlying resistance to multiple HG types of soybean cyst nematode in soybean PI 437655. *Theor. Appl. Genet.* **128**, 15–23 (2015).
2. Chang, S. J. C. *et al.* Association of loci underlying field resistance to soybean sudden death syndrome (SDS) and cyst nematode (SCN) race 3. *Crop Sci.* **37**, 965–971 (1997).
3. Williams, B. *et al.* Genetic Analysis of Root and Shoot Traits in the 'Essex' By 'Forrest' Recombinant Inbred Line (RIL) Population of Soybean [Glycine max (L.) Merr.]. *J. Plant Genome Sci.* **1**, 1–9 (2012).
4. Wu, X. *et al.* QTL, additive and epistatic effects for SCN resistance in PI 437654. *Theor. Appl. Genet.* **118**, 1093–1105 (2009).
